# Lymph node extracellular matrix skews the immune function of human fibroblastic reticular cells

**DOI:** 10.1016/j.isci.2026.117516

**Published:** 2026-09-16

**Authors:** Daphne Panocha, Janna E.G. Roet, Michael de Kok, Tanja Konijn, Henk P. Roest, Luc J.W. van der Laan, Charlotte M. de Winde, Reina E. Mebius

**Affiliations:** 1 Amsterdam UMC Location Vrije Universiteit Amsterdam, Department of Molecular Cell Biology & Immunology, De Boelelaan 1117, 1081 HZ Amsterdam, the Netherlands; 2 Amsterdam Institute for Immunology and Infectious Diseases, Amsterdam, the Netherlands; 3 Cancer Center Amsterdam, Cancer Biology & Immunology, Amsterdam, the Netherlands; 4 Erasmus MC Transplant Institute, University Medical Center Rotterdam, Department of Surgery, Dr. Molewaterplein 40, 3015 GD Rotterdam, the Netherlands

**Keywords:** lymph node, fibroblastic reticular cell, T cell, extracellular matrix, laminin, adaptive immunity

## Abstract

Lymph node (LN) fibroblastic reticular cells (FRCs) are key immunomodulators that regulate adaptive immune responses and provide structural support via extracellular matrix (ECM) production. However, how ECM composition shapes FRC-immune cell interactions remains unclear. Here, we show that ECM composition, specifically collagen type I, laminin α4, or laminin α5, critically determines the immunological status of human FRCs. Bulk RNA sequencing shows that FRCs grown on collagen closely resemble those in native human LN ECM, whereas laminin α4 and α5 differentially skew FRC transcriptomes toward homeostatic/tolerogenic or pro-inflammatory profiles, respectively. These ECM-imprinted FRC states subsequently shape distinct CD4^+^ T cell fates in autologous co-culture assays: laminin α4-FRCs maintain tissue-resident T cells, whereas laminin α5-FRCs selectively enhance effector T cell proliferation. Together, these findings identify ECM as an active instructive signal that, through FRCs, orchestrates immune cell behavior and offers a tunable axis for therapeutic immune modulation.

## Highlights

• Collagen-cultured human FRCs closely resemble native lymph node ECM-cultured FRCs
• Laminin α4 imprints FRCs with homeostatic, tolerogenic transcriptional profiles
• Laminin α5 skews FRCs toward pro-inflammatory transcriptional profiles
• ECM-imprinted FRCs differentially control CD4^+^ T cell residency and proliferation

## Graphical abstract

**Figure:**
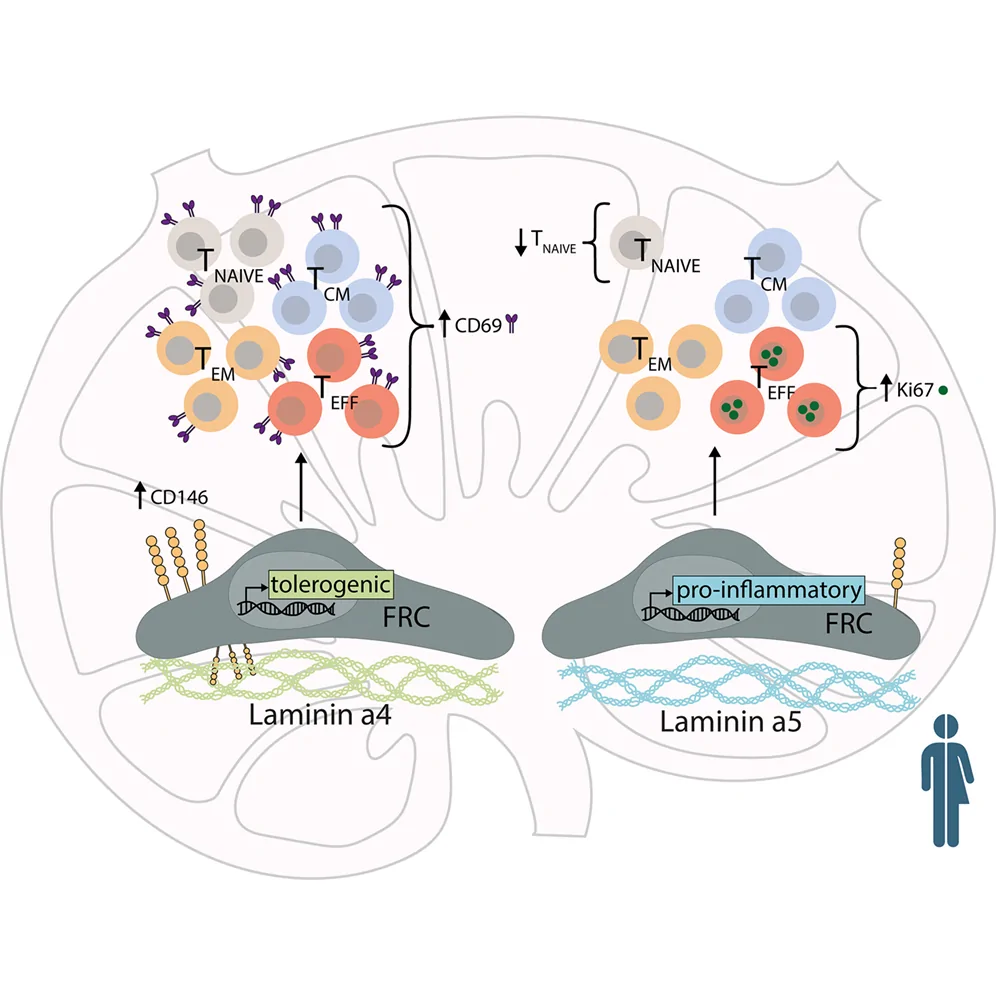

## Teaser

Immunology; Cell biology

## Introduction

Lymph nodes (LNs) are complex tissue-draining organs, which filter lymph and serve as the primary sites for the efficient initiation of adaptive immune responses.^1^ To orchestrate this, LNs have a specialized architecture that compartmentalizes lymphocytes into B- and T cell areas, while simultaneously promoting directed migration and interaction of various immune cells.^1^ This overall architecture is formed by distinct types of LN stromal cells (LNSCs): lymphatic endothelial cells (LECs), blood endothelial cells (BECs) and fibroblastic reticular cells (FRCs). Transcriptomic analyses have revealed cellular heterogeneity within these stromal cells, with specific locations and functions for each subset within the LN.^2^^,^^3^^,^^4^^,^^5^^,^^6^ FRCs form the main subset of LNSCs and support the LN architecture by producing various extracellular matrix (ECM) proteins.^7^

ECM is composed of a variety of proteins, which can be divided into three core matrisome subsets: collagens, proteoglycans, and glycoproteins.^8^ Collagens form fibrils to provide structural strength, and proteoglycans are integrated within these collagen fibrils. Through their binding to collagens as well as their ability to bind to glycoproteins and capture growth factors, proteoglycans contribute to the regulation of protein complexes in the ECM. Glycoproteins, in turn, have various functions, including interactions to allow ECM assembly, promoting cell adhesion and cell signaling as well as binding of growth factors.^8^ The ECM in resting human LNs is mostly composed of collagens, especially collagen type 1, followed by glycoproteins and proteoglycans, as identified by mass spectrometry of decellularized human LNs.^9^Collagen-type 1, often used for *in vitro* cell culture, resulted in the outgrowth of various human FRC subsets, although not all subsets identified *ex vivo* were also preserved *in vitro.*^10^^,^^11^

Using high-resolution light microscopy and 3D reconstruction, Sixt et al.^12^ were among the first to characterize LN ECM and elucidate its specialized structural organization, called the conduit system.^12^^,^^13^ In LN T cell areas, the ECM produced by FRCs establishes this conduit system that consists of various highly organized ECM components of all three core matrisome subsets, forming a cable-like structure known as the central fibrillar core, which transports and filters lymph fluid and is surrounded by a microfibrillar zone and a basement membrane to which the FRCs are attached. While abundantly present in T cell areas, conduits in B cell areas are sparse and poorly branched.^7^ The outer layer of the conduit system, the basement membrane, is enwrapped by FRCs, together forming the reticular network.^12^^,^^13^

LNs are important sites of immune regulation. The reticular network is quick to respond to the inflammatory state of the LN, permitting LN swelling during immune responses, while supporting a contractile state of the LN during homeostasis.^13^^,^^14^^,^^15^^,^^16^^,^^17^^,^^18^ These dynamics are accompanied by alterations in ECM production and deposition by FRCs.^7^^,^^13^^,^^16^ FRCs simultaneously steer T cell immunity depending on the inflammatory state of an LN. While FRCs during an immune response allow T cell activation and control their proliferation capacity,^11^ they promote a tolerant LN microenvironment during homeostasis via secretion of soluble factors, FRC surface marker expression of immunomodulatory molecules, and self-antigen presentation via HLA-DR.^7^^,^^19^^,^^20^

Laminins, a family of glycoproteins, are functional ECM components of the conduit basement membrane, and different laminins are associated with the activation state of the LN.^21^^,^^22^^,^^23^^,^^24^^,^^25^^,^^26^ Laminins consist of an α-, β-, and γ-chain, of which the α-chain allows cell adhesion with the enveloping FRCs. Interestingly, the ratio of two laminin forms, laminin α4 and laminin α5, differs in resting versus activated LNs in mice.^21^^,^^22^^,^^24^^,^^25^ Under tolerogenic conditions, mainly laminin α4 is expressed, while inflammation-induced increased expression of laminin α5 and reduce laminin α4.^21^^,^^22^ Due to these differences, laminin α4 is correlated with tolerance, while laminin α5 is correlated with immunity.^21^^,^^22^^,^^24^^,^^25^ This is further supported by *in vitro* mouse studies in which laminin α4 diminished CD4^+^ T cell activation and promoted regulatory T cell (Treg) polarization, while laminin α5 supported mouse CD4^+^ T cell activation, proliferation, and differentiation toward effector T cells^21^^,^^22^^,^^24^**.** However, in the above-mentioned studies, the direct effect of laminins on T cells was examined. Yet, since FRCs enwrap the conduit system and are thus in direct contact with the ECM proteins of the basement membrane, they occupy a strategic position in which they prevent direct and prolonged contact between ECM proteins and freely moving T cells in LNs. As a consequence, T cells predominantly interact with the surface of FRCs. This spatial arrangement implies that ECM-derived signals are likely perceived and integrated by FRCs and subsequently conveyed to T cells, suggesting a triadic mode of communication between ECM, FRCs and T cells in LNs.^7^ Based on this, together with the multifaceted functions of FRCs in T cell immunity, we hypothesize that laminins in the basement membrane of the conduit system may also directly influence FRC immune function, which in turn affects T cell dynamics.

Here, we verified the critical role of ECM in FRC and T cell immunological status in LNs. We specifically focused on the basement membrane components laminin α4 and laminin α5 due to their roles in steering the LN microenvironment. Collagen type I, widely used in cell culture, was included as a control. We identified that FRCs grown on collagen transcriptionally resemble FRCs grown on native human LN ECM, while FRCs grown on recombinant laminin α4 or laminin α5 have different transcriptomic profiles, with laminin α4 inducing a more homeostatic FRC profile compared to a pro-inflammatory FRC profile on laminin α5. The different ECM proteins also resulted in differences in FRC surface markers, as laminin α4 showed an increase in HLA-DR and CD146 (MCAM) expression on FRCs. We further elaborated the role of ECM by co-culturing FRCs and CD4^+^ T cells on different ECM coatings and examining the effect on T cell subsets in the absence of any stimulus. Co-cultures enriched with FRCs demonstrated a decrease in naive CD4^+^ T cells in laminin α5-FRC-enriched co-cultures, while in this condition an increase in the proliferation marker Ki67 in effector subsets was observed. Furthermore, laminin α4-FRCs induced CD69 expression on CD4^+^ T cells, suggesting that laminin α4-FRCs promote tissue residency within LNs. Overall, this study unveiled the importance of ECM, and specifically laminins, in organizing the LN microenvironments via their direct effect on FRCs and subsequently T cells.

## Results

### Collagen-cultured human FRCs have a similar transcriptome to FRCs grown on native human lymph node ECM

**Figure 1:**
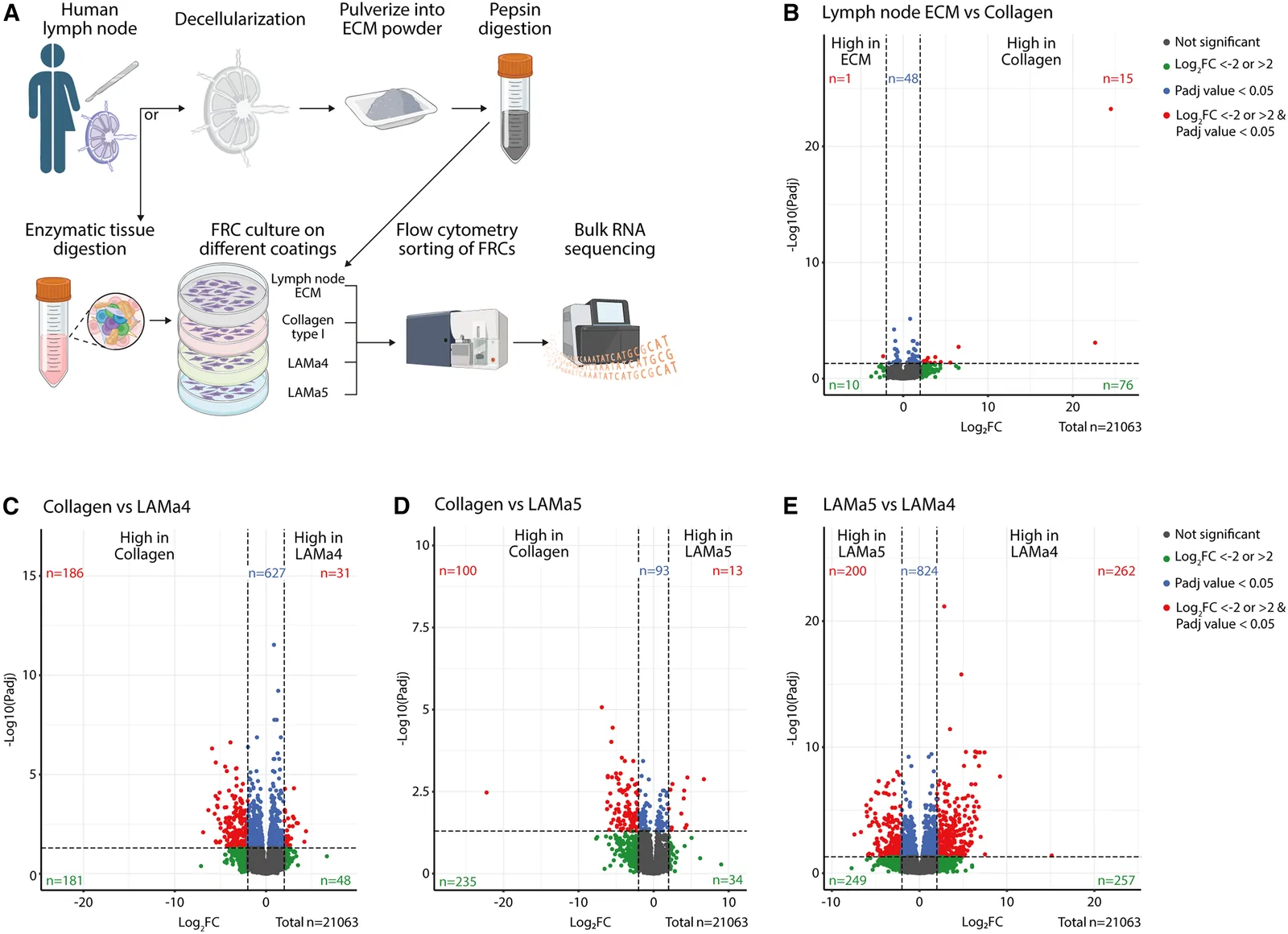
Transcriptomes of human FRCs grown on various ECM coatings (A) Schematic representation of the experimental setup to investigate the transcriptomes of human FRCs grown on various ECM coatings: native lymph node ECM, collagen (collagen type 1), laminin α4, and laminin α5. (B–E) Volcano plots show changes in gene expression of FRCs grown on lymph node ECM vs. collagen (B), collagen vs. laminin α4 (C), collagen vs. laminin α5 (D), and laminin α5 vs. laminin α4 (E). Total number of genes measured: 21063. Visualized are the gene numbers with Log_2_FC < −2 or >2 (green), Padj value <0,05 (blue), or both Log_2_FC < −2 or >2 and Padj value <0,05 (red). Differential gene expression was analyzed via DESeq2 with the Wald test. Data are from bulk RNA-seq samples of FRCs between passages 1–4, derived from donors 1, 2, and 3 (LN ECM: *n* = 6; collagen: *n* = 6; laminin α4: *n* = 6; laminin α5: *n* = 4). ECM: extracellular matrix, FRCs: fibroblastic reticular cells, LAMa4/5: laminin α4/5, Log_2_FC: log_2_ fold change, n: number of genes, Padj: adjusted *p* value. See also Figure S1 and Table S3.

While the human LN ECM consists of various collagens, glycoproteins, and proteoglycans, collagen is most abundant and forms the main component of LN ECM.^9^ Although human LN ECM would be a more physiologically relevant surface for culturing human FRCs, the isolation of human LN ECM requires the use of sparsely available healthy human LNs. For this reason, we have recently used collagen as an alternative for human LN ECM and characterized human FRCs from healthy LN cell suspensions grown on collagen based on expression of surface markers.^10^ To address whether FRCs grown on collagen-coated surfaces represent FRCs grown on ECM isolated from decellularized human LNs, we performed bulk RNA sequencing (RNAseq) (Figure 1A).^9^^,^^27^^,^^28^^,^^29^ FRCs from healthy human LNs were cultured on different ECM coatings for 1–4 passages, and sorted FRCs were used for bulk RNA-seq (Figure 1A). When comparing the transcriptomes of FRCs grown on LN ECM versus collagen, a total of 21063 genes were detected, of which only 64 genes were significantly differentially expressed between these two ECM coatings (Figure 1B). Moreover, only 16 out of these 64 genes had at least a log_2_ fold change difference of 2 (Figure 1B), indicating that the transcriptomes of human FRCs grown on collagen are highly similar to FRCs grown on human lymph node ECM. Thus, based on the transcriptional profiles, collagen coating is a physiologically relevant substitute for human LN ECM coating for *in vitro* culture of human FRCs.

### Collagen, laminin α4, or laminin α5 induce different transcriptomic profiles in cultured human FRCs

Next to collagen, native LN ECM contains various other ECM proteins, which might affect LN function.^9^ Especially the ratio of two subtypes of laminins, laminin α4 and laminin α5, which are both abundantly present in resting human LNs,^9^ have been described to differ in resting versus activated LNs in mice, as laminin α4 is increased under tolerogenic conditions while laminin α5 is increased upon inflammation.^21^^,^^22^^,^^24^^,^^25^ Here, we addressed whether laminin α4 and laminin α5 could directly influence the transcriptome of human FRCs.

To check whether our cultured FRCs represent FRCs in a homeostatic human lymph node, we compared our bulk RNA-seq with a publicly available scRNA-seq dataset of human FRCs from homeostatic lymph nodes (Figure S1).^30^ This comparison shows that approximately 70% of the top 10 differentially expressed genes from the published human scRNA-seq dataset are present in human FRCs cultured on the different ECM coatings. This indicates that the cultured FRCs retain substantial transcriptional overlap with *ex vivo* human FRC subsets, rather than an unrelated fibroblast profile. Simultaneously, the overlap is incomplete, and subset-specific signatures are averaged out by bulk sequencing. Thus, the cultured FRCs can be viewed as FRC-like states, rather than exact equivalents of all *in vivo* FRC subsets. Notably, genes characteristic of the B cell cluster (cluster 9) in the publicly available dataset were largely absent in the cultured FRC bulk RNA-seq data, indicating the specificity of the comparison of the RNA-seq data with the scRNA-seq dataset of human FRCs. Within the transcriptomes of FRCs grown on collagen, laminin α4, or laminin α5, a total of 21063 genes were detected (Figures 1C–1E). When comparing FRCs grown on collagen versus laminin α4, expression of 844 genes significantly differed, of which 217 genes had at least a log_2_ fold change difference of 2 (Figure 1C). In the comparison of FRCs grown on collagen versus laminin α5, 206 genes were significantly differentially expressed, of which 113 genes had at least a log_2_ fold change difference of 2 (Figure 1D). The most significantly differentially expressed genes (DEGs) (*n* = 1286) were detected when comparing FRCs grown on laminin α5 versus laminin α4, of which 462 genes had at least a log_2_ fold change difference of 2 (Figure 1E). These data show that ECM provided to FRCs *in vitro* affects their transcriptomic profiles.

**Figure 2:**
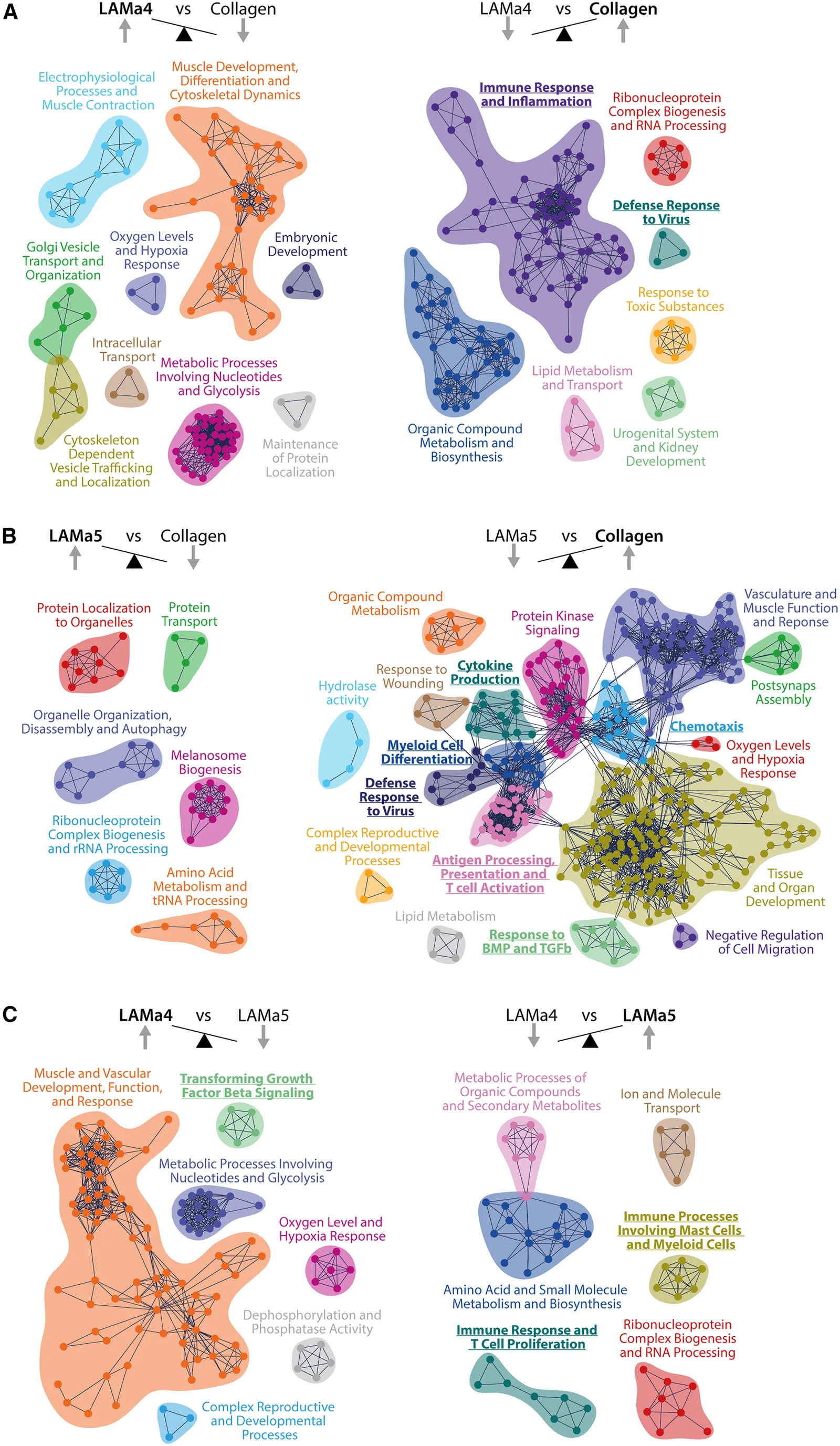
Different gene set clusters enriched in FRCs grown on collagen, laminin α4, and laminin α5 (A–C) Visualization of enriched gene set clusters identified in FRCs grown on laminin α4 vs. collagen (A), laminin α5 vs. collagen (B), and laminin α4 vs. laminin α5 (C). For each comparison, a gene set enrichment analysis was performed based on the differentially expressed genes, followed by gene set cluster analysis of the significantly enriched gene sets (*Q* value <0.04). Clusters were annotated based on the gene sets within the cluster. See Figure S2 for gene set dotplots from a selection of clusters. Data are from bulk RNA-seq samples of FRCs between passages 1–4, derived from donors 1, 2, and 3 (collagen: *n* = 6; laminin α4: *n* = 6; laminin α5: *n* = 4). LAMa4/5: laminin α4/5. See also Figure S2.

To further analyze these different transcriptomic profiles, we performed gene set enrichment analysis followed by gene set cluster analysis on the transcriptome data of FRCs grown on laminin α4 versus collagen (Figure 2A), laminin α5 versus collagen (Figure 2B), and laminin α4 versus laminin α5 (Figure 2C). The gene sets within the clusters were used for annotation of the clusters. When comparing FRCs grown on laminin α4 versus collagen, the gene set clusters enriched in laminin α4 include muscle and cytoskeletal clusters as well as hypoxia response and glycolytic metabolic processes, while the gene set clusters enriched in collagen include immune responses, inflammation, RNA processing, and lipid and organic compound metabolism (Figures 2A and S2A; Table S1). For the comparison of FRCs grown on laminin α5 versus collagen, the gene set clusters enriched for laminin α5 include transfer RNA (tRNA) and ribosomal RNA (rRNA) processing, as well as protein localization and organelle organization, and the gene set clusters enriched for collagen include cytokine production, chemotaxis, antigen presentation, T cell activation, myeloid cell differentiation, response to BMP and TGFβ and tissue development (Figures 2B and S2B; Table S1). When comparing FRCs grown on laminin α4 versus laminin α5, the gene set clusters enriched for laminin α4 include a muscle and vasculature cluster, TGFβ signaling as well as hypoxia response and glycolytic metabolic processes, while the clusters enriched for laminin α5 include immune processes involving mast cells, myeloid cells and T cell proliferation, as well as RNA processing (Figures 2C and S2C; Table S1).

Overall, these data indicate that FRCs grown on different ECM components have different transcriptomic profiles related to lymph node immune function: the gene set clusters enriched in collagen associate with immune responses and inflammation; the gene set clusters enriched in laminin α4 associate with TGFβ signaling; and the gene set clusters enriched in laminin α5 associate with immune responses and T cell proliferation.

### The type of ECM coating affects gene expression of immune-controlling factors in FRCs

**Figure 3:**
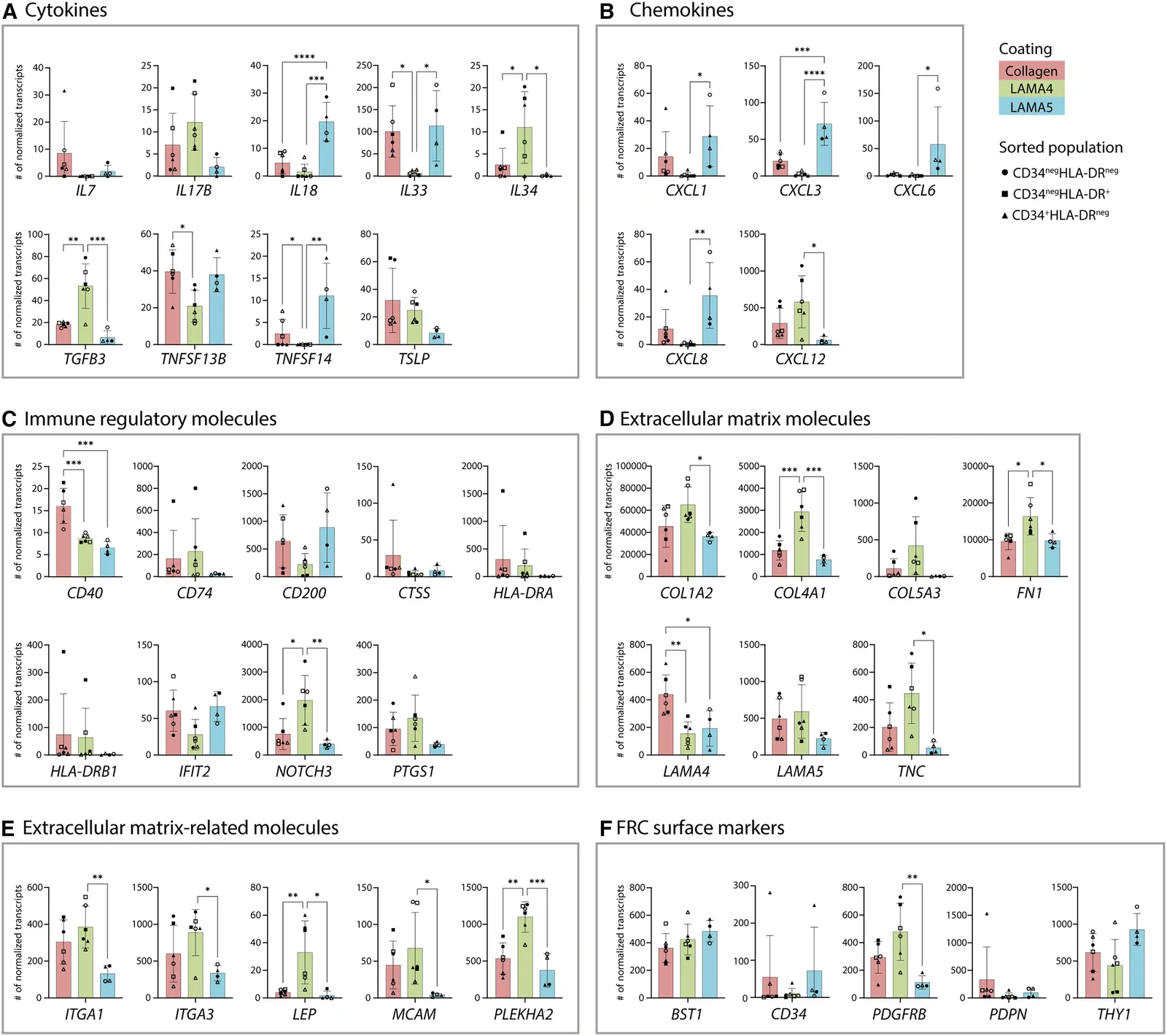
The type of ECM coating affects FRC gene expression of cytokines, chemokines, immune regulatory molecules, ECM molecules, ECM-related molecules, and FRC surface markers The number of normalized transcript counts per selected gene is visualized for FRCs grown on laminin α4, laminin α5, or collagen. The genes for cytokines (A), chemokines (B), immune regulatory molecules (C), extracellular matrix molecules (D), extracellular matrix-related molecules (E), and FRC surface markers (F) were selected based on the differentially expressed genes (Padj <0.05) in at least one of the three following comparisons: laminin α4 vs. collagen, laminin α5 vs. collagen or laminin α4 vs. laminin α5 (see Table S2). *CD200*, *BST1*, *CD34* and *THY1* were added for additional characterization of FRCs. The different symbol shapes represent the sorted populations of FRCs between passages 1–4 (open = donor 1; filled symbol = donor 2 for collagen and laminin α4, or donor 3 for laminin α5). *N* = 6 bulk RNA-seq samples for collagen and laminin α4, and *n* = 4 bulk RNA-seq samples for laminin α5. Data are represented as mean ± SD. One-way ANOVA with Tukey’s multiple comparisons test.∗: *p* < 0.05; ∗∗: *p* < 0.01; ∗∗∗: *p* < 0.001; and ∗∗∗∗:*p* < 0.0001. See also Table S2.

To investigate specific gene differences between FRCs grown on collagen, laminin α4, or laminin α5, we analyzed differentially expressed genes coding for cytokines, chemokines, immune regulatory molecules, ECM and ECM-related proteins as well as FRC surface markers (Table S2). The normalized transcript counts of these genes were analyzed between FRCs grown on collagen, laminin α4, and laminin α5 (Figure 3).

FRCs grown on laminin α4 had decreased gene expression of the IL-1 family cytokine and alarmin *IL33*,^31^ and increased gene expression of the pleiotropic cytokine *IL34*^32^ and immunosuppressive cytokine *TGFB3*,^11^ as compared to laminin α5 and collagen (Figure 3A). FRCs grown on laminin α5 had increased expression of the IL-1 family pro-inflammatory cytokine *IL18*^33^ and pleiotropic cytokine *TNFSF14* (LIGHT),^34^^,^^35^ as compared to laminin α4 and collagen (Figure 3A). FRCs grown on collagen had increased expression of the B-cell survival factor *TNFSF13B* (BAFF) as compared to laminin α4. The transcript counts for the cytokines *IL7*, *IL17B* and *TSLP* were not significantly different between the ECM coatings (Figure 3A). Furthermore, the inflammatory chemokines *CXCL1*, *CXCL3*, *CXCL6* and *CXCL8* were enriched in FRCs grown on laminin α5 as compared to laminin α4, while the homeostatic chemokine *CXCL12*,^36^ which is associated with immune tolerance induction by FRCs,^37^ was enriched in FRCs grown on laminin α4 as compared to laminin α5 (Figure 3B).

Gene expression of the co-stimulatory molecule *CD40* was enriched in FRCs grown on collagen, while there was no difference for the other antigen-presenting-related molecules: *CD74*, the invariant chain; *CTSS*, cathepsin S for antigen processing; *HLA-DRA* and *HLA-DRB1*, the genes for MHC class II (Figure 3C). FRCs, independent of ECM coating, expressed genes for the immune inhibitory molecule *CD200*,^38^ the interferon-stimulated antiviral molecule *IFIT2*, and *PTGS1* (COX1), which is expressed by FRCs to constrain the proliferation of T cells^11^ (Figure 3C). *NOTCH3* expression was enhanced in FRCs grown on laminin α4, which is essential for organ and tissue development and homeostasis, as well as collagen production in mesenchymal cells.^39^^,^^40^

In addition, gene expression of most ECM molecules was enriched in FRCs grown on laminin α4, including collagen type 1 and 4 (*COL1A2* and *COL4A1*), fibronectin 1 (*FN1*), and tenascin C (*TNC*) (Figure 3D). Interestingly, only gene expression of laminin α4 (*LAMA4)* was enriched in FRCs grown on collagen as compared to laminin α4 or laminin α5 (Figure 3D). Moreover, gene expression of ECM-related molecules was also enriched in FRCs grown on laminin α4 (Figure 3E). These include integrin alpha 1 (*ITGA1*), a collagen receptor^8^; integrin alpha 3 (*ITGA3*), a laminin receptor^41^; Leptin (*LEP*), associated with ECM production in FRCs^7^; *MCAM* (CD146), a laminin receptor^41^; *PLEKHA2*, a regulator of cell adhesion to ECM proteins.^42^ Lastly, most FRC surface markers (*BST1*, *CD34*, *PDPN*, *THY1*/*CD90*) were not differently expressed by FRCs grown on different ECM coatings, except for *PDGFRB*, which was enhanced in FRCs grown on laminin α4 as compared to laminin α5 (Figure 3F).

To validate our FRC bulk RNA-seq data, we cross-checked our data with a publicly available human lymph node single-cell RNA-seq dataset.^43^ We compared the significantly expressed DEGs in the non-hematopoietic stromal cells between activated and control LN slices from Fergusson et al.^43^ with the DEGs per ECM comparison from our bulk RNA-seq, and visualized the genes enriched in both datasets (Table S3). We found that the DEGs enriched in activated human LN slices^43^ were also predominantly enriched in collagen- or laminin α5-cultured FRCs. Conversely, the DEGs enriched in control human LN slices^43^ were substantially enriched in laminin α4-cultured FRCs. This suggests that collagen- or laminin α5-cultured FRCs associate with an activated LN, while laminin α4-cultured FRCs align with homeostatic LNs.

To conclude, FRCs grown on laminin α4 show a tolerogenic transcriptional profile with lower gene expression of pro-inflammatory cytokines and higher gene expression of immune homeostatic cytokines and chemokines, as well as ECM proteins and ECM-binding molecules. In contrast, FRCs grown on laminin α5 have increased gene expression of pro-inflammatory cytokines and chemokines, contributing to the pro-inflammatory transcriptional profile of these FRCs.

### Laminin α4 induces CD146 expression on human FRCs and colocalizes with CD146 in human lymph nodes

**Figure 4:**
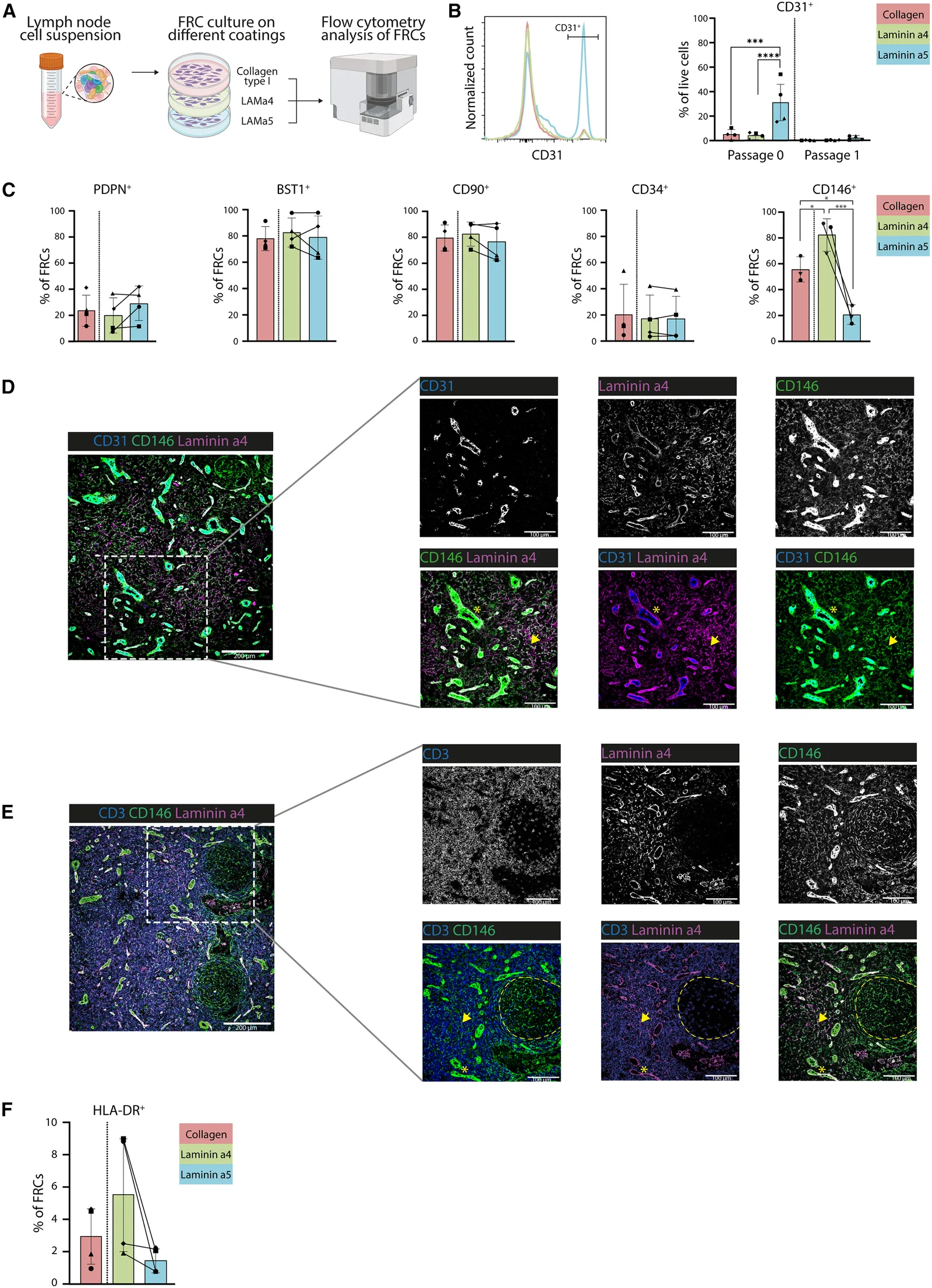
Characterization of human FRC surface markers on collagen, laminin α4 and laminin α5 (A) Schematic representation of the experimental setup to investigate the surface markers of human FRCs grown on various ECM coatings: collagen, laminin α4 and laminin α5. (B) Histogram of CD31 normalized counts and percentages of CD31^+^ cells in passage 0 and 1 (*n* = 4 donors). (C) Percentages of PDPN^+^, BST1^+^, CD90^+^, CD34^+^ FRCs (*n* = 4 donors), and CD146^+^ FRCs (*n* = 3 donors) on the different ECM coatings from passage 1. (D) Confocal images of human lymph node (donor 8) for CD31 (blue), laminin α4 (magenta), and CD146 (green). (E) Confocal images of human lymph node (donor 8) for CD3 (blue), laminin α4 (magenta) and CD146 (green). Yellow arrow indicates reticular fibers; yellow asterisk indicates vessels. (F) Percentages of HLA-DR^+^ FRCs on different ECM coatings from passage 1 (*n* = 4 donors). The different symbol shapes represent different donors: circle = donor 1, square = donor 4, triangle = donor 5, reverse triangle = donor 2, diamond = donor 6, hexagon = donor 7. Confocal images of (D and E) are representative images of one from six different human lymph node donors. Scale bars represent 200 μm in the overview images and 100 μm in the magnified images. Data are represented as mean ± SD. Two-way ANOVA (A) or one-way ANOVA (C and F) with Tukey’s multiple comparisons test. ∗: *p* < 0.05; ∗∗∗: *p* < 0.001; and ∗∗∗∗:*p* < 0.0001. See also Figures S4 and S5.

To determine whether the different ECM coatings not only induce changes at transcriptomic levels but also lead to changes in cell surface expression of FRC-specific molecules, we cultured human LN suspensions on collagen, laminin α4, and laminin α5 to grow out FRCs, which were subsequently analyzed using spectral flow cytometry (Figures 4A and S4). In passage 0 of FRCs cultured on different types of ECM, a significant presence of endothelial stromal cells was detected in laminin α5 cultures compared to collagen and laminin α4 (Figure 4B). However, further passaging of FRCs resulted in the loss of endothelial cells in all three ECM cultures (Figure 4B).

Next, we analyzed the levels of classical FRC soluble factors in the culture supernatants and could detect BAFF, CXCL12, and IL-7 (Figure S5). Although CXCL12 levels seem to be highest in the laminin α4-FRC supernatant (similar to the gene expression levels, Figure 3B), no significant changes could be detected between the different ECM-cultured FRCs (Figure S5). The lack of detectable levels of CXCL13, CCL19, and CCL21 at both transcriptomic and protein levels (Figures 3 and S5) could be due to the lack of fluid flow, which is required for the induction of chemokines in FRCs.^44^^,^^45^^,^^46^

Further characterization of the FRC surface marker profile showed that the transmembrane glycoprotein podoplanin (PDPN), involved in both homeostatic and inflammatory processes,^7^ was not significantly different between FRCs cultured on either collagen, laminin α4, or laminin α5 (Figure 4C). For BST1,^47^ CD90^6^^,^^48^ and CD34^10^ expressing FRCs, which are FRC surface markers described for multiple FRC subsets with different locations and functions within the LN, no striking differences were detected between collagen, laminin α4 and laminin α5 cultures (Figure 4C). Interestingly, a significantly higher percentage of FRCs expressed CD146 when cultured on laminin α4 compared to collagen and laminin α5 (Figure 4C). CD146 is a highly glycosylated type I transmembrane protein that is identified as a receptor for laminin α4.^49^^,^^50^ These findings are in line with the gene expression levels shown in Figure 3F.

Since laminin α4 cultures result in more CD146^+^ FRCs, we investigated the spatial organization of laminin α4 and CD146 in human LNs using confocal microscopy. We combined laminin α4 and CD146 staining with CD31 or CD3 to respectively identify endothelial vessels or the T cell zone within human LNs (Figures 4D and 4E). Laminin α4 was highly expressed around CD31^+^ vessels, but did not overlap with CD31, and was also detected in the surrounding tissue (Figure 4D). Indeed, laminin α4 expression was dense within the CD3^+^ T cell zone, and sparse within B cell follicles (Figure 4E). CD146, also a known endothelial cell marker,^50^^,^^51^ overlapped with CD31^+^ endothelial cells and colocalized with laminin α4 around the vessel (Figure 4D). Furthermore, CD146 was also present in both T cell zones and B cell follicles, showing a reticular fiber pattern colocalizing with laminin α4 (Figure 4E).

Collectively, these data show that laminin α4 increases the laminin α4-binding receptor CD146 on FRCs, and that laminin α4 and CD146 are in close proximity in the T cell zone of human lymph nodes.

### ECM-imprinted FRCs affect lymph node T cell subsets

Our finding that laminin α4 is in close spatial proximity to its receptor CD146 on FRCs within the T cell zone of human LNs suggests a potential role for laminin α4 in regulating T cell-stromal interactions. Previous studies have demonstrated that laminin α4 contributes to the induction of immune tolerance by promoting Treg differentiation in mouse LNs.^21^^,^^23^^,^^24^ In human lymph nodes, the MHC-II molecule HLA-DR has recently been shown to contribute to regulatory T cell (Treg) maintenance and peripheral tolerance.^20^^,^^52^ Based on these observations, we investigated HLA-DR expression on human FRCs cultured on different ECM coatings. FRCs showed the expression of HLA-DR on their cell surface when cultured on either collagen, laminin α4, or laminin α5, with the highest expression level in laminin α4-cultured FRCs (Figure 4F). This suggests that laminin α4 in human LNs contributes to Treg maintenance via the expression of HLA-DR on FRCs, possibly contributing to immune tolerance.

**Figure 5:**
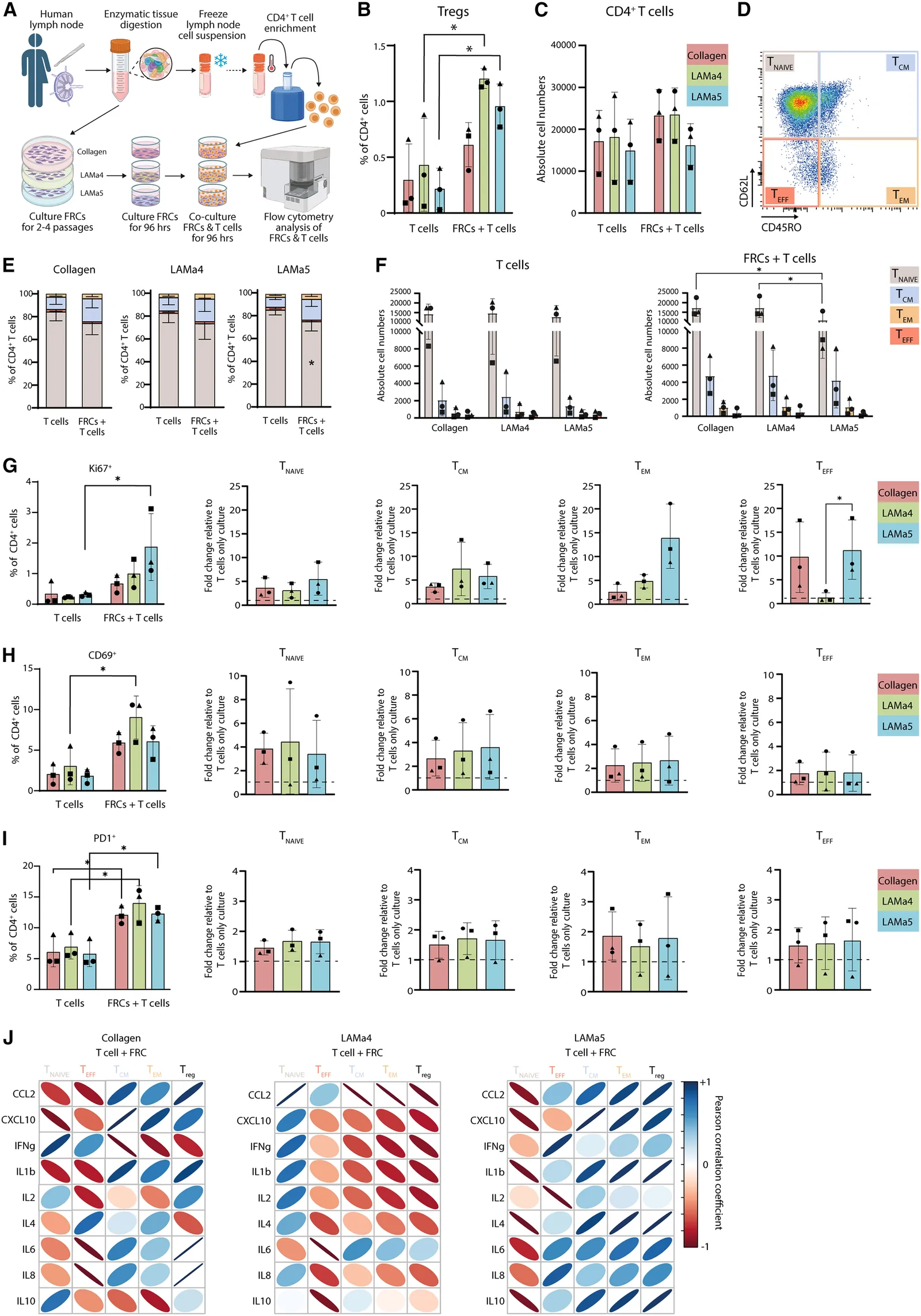
ECM-imprinted FRCs affect lymph node T cell subsets (A) Schematic representation of the experimental setup to investigate the T cell phenotype in co-culture with FRCs grown on various ECM coatings: collagen (collagen type 1), laminin α4 (LAMa4) and laminin α5 (LAMa5). CD4^+^ T cell purity (Table S3) and phenotype were assessed by spectral flow cytometry. A representative gating strategy can be found in Figure S6. (B) Percentage of CD4^+^CD25^+^CD127^low^FoxP3^high^ Tregs after 96 h co-culture. (C) Absolute cell numbers of CD4^+^ T cells. (D) Gating strategy of CD4^+^ subsets. (E) Average proportion of CD4^+^ subsets in culture ± FRCs. (F) Absolute cell numbers of CD4^+^ subsets on various ECM coatings. (G–I) Percentage of Ki67^+^ (G), CD69^+^ (H), or PD1^+^ (I) CD4^+^ T cells in culture ± FRCs and relative fold change in percentage Ki67^+^, CD69^+^, or PD1^+^ cells per T cell subset in either collagen, laminin α4, or laminin α5 cultures enriched with FRCs compared to T cell-only cultures, which are set to 1. (J) Pearson’s correlation plots of T cell subsets, cytokines, and chemokines in FRC + CD4^+^ T cell co-cultures per ECM coating. *N* = 3 donors. Each datapoint represents one donor (mean of technical duplicates); symbol shapes indicate donors: square = donor 2, circle = donor 4, triangle = donor 7. FRCs within the co-culture are at passage 3 (donor 4 and 7) or passage 4 (donor 2). All data are presented as mean ± SD. Two-way ANOVA with Tukey’s multiple comparisons test.∗: *p* < 0.05. See also Figures S6–S9 and Table S4.

Therefore, we next investigated the presence of Tregs in FRC-T cell co-cultures. Here, we performed co-culture experiments of confluent FRCs, expanded on either collagen, laminin α4, and laminin α5, with autologous CD4^+^ T cells, in the absence of an external stimulus, and compared them to CD4^+^ T cells cultured on the same ECM (Figure 5A). In line with previously reported findings,^20^ FRC co-culture with T cells significantly increased the percentage of HLA-DR^+^ FRCs compared to FRC cultures without T cells (Figure S7A). Based on the literature,^53^^,^^54^^,^^55^ we gated the functionally distinct population of Tregs as CD4^+^CD25^+^CD127_low_FoxP3^high^ cells (Figure S6). The FRCs in all ECM conditions were able to maintain a Treg population (Figure 5B). Interestingly, only laminin α4 and laminin α5 significantly increased the percentage of Tregs upon FRC co-culture (Figure 5B).

Since we did not find a significant difference in the percentage of Tregs between laminin α4 and laminin α5 FRC co-cultures, we next addressed whether additional T cell subsets were affected by the different ECM-imprinted FRCs. We therefore investigated the presence of naive (T_NAIVE_; CD62L^+^CD45RO^−^), central memory (T_CM_; CD62L^+^CD45RO^+^), effector memory (T_EM_; CD62L^−^CD45RO^+^) and effector (T_EFF_; CD62L^−^CD45RO^−^) T cells (Figures 5C–5F). Overall, the addition of FRCs to the co-cultures resulted in more CD4^+^ T cells at the time of analysis (Figure 5C), of which naive CD4^+^ T cells were the largest proportion (Figures 5E and 5F). Interestingly, the addition of collagen, laminin α4, or laminin α5 FRCs led to a relative reduction of the T_NAIVE_ population, although only with a significant effect in the laminin α5 co-cultures compared to the T cells cultured without FRCs (Figure 5E). Similarly, laminin α5-FRCs also induced significantly lower absolute numbers of naive T cells compared to collagen- and laminin α4-FRCs (Figure 5F). The addition of FRCs to the T cells resulted in a relative increase in the number of T_CM_ cells, the second largest population, and, to a lesser extent, the T_EM_ cells (Figures 5E and 5F). Conversely, in the presence of FRCs, the small proportion of T_EFF_ cells became even smaller (Figures 5E and 5F). Thus, FRCs change the ratio between naive and memory/effector CD4^+^ T cells by favoring the induction of memory T cell populations while lowering the proportion of effector T cells.

To determine whether the increase of Tregs in laminin α4 and laminin α5 FRC-T cell co-cultures (Figure 5B) was reflected by a functional control of T cells, we next analyzed the proportion of CD4^+^ T cells expressing the proliferation marker Ki67.^56^^,^^57^^,^^58^ Remarkably, a significant increase in the percentage of Ki67-expressing T cells was seen in the presence of laminin α5-cultured FRCs, and not in the presence of collagen- and laminin α4-cultured FRCs (Figure 5G). This difference was in particular caused by a significant increase in Ki67^+^ T_EFF_ cells on laminin α5-FRCs compared to laminin α4-FRCs, which showed an unaltered fold change in Ki67^+^ T_EFF_ cells (Figure 5G).

Further analysis of T cell markers showed a significant increase of the putative tissue residency marker CD69 on CD4^+^ T cells in laminin α4-FRC enriched co-cultures (Figure 5H) as well as an increase of the inhibitory receptor PD1 in co-cultures enriched with the 3 different types of ECM FRCs (Figure 5I), while no differences were observed for the late T cell activation markers CD25 and HLA-DR, the immune checkpoint CTLA4, homing molecule CCR7, and co-stimulatory receptor CD27 (Figure S8).

As FRCs and T cells communicate via both receptor-ligand interactions and soluble factors, we next elucidated the role of soluble factors in FRC -T cell crosstalk. Therefore, we analyzed cell culture supernatants for the inflammatory cytokines and chemokines CCL2, CXCL10, IFNγ, IL1b, IL2, IL4, IL6, IL8, and IL10 (Figure S9). No significant differences between the ECM coatings could be detected, which is likely due to donor variation. To overcome this variation, we analyzed the soluble factor data by making use of correlation analysis, in which correlation coefficients or patterns are analyzed rather than raw data values in order to overcome the skewing by donor variation. This revealed that in laminin α4-FRC co-cultures, the proportion of differentiated T cells correlated negatively with inflammatory cytokines and chemokines, while naive T cells showed a positive correlation, suggesting a suppressive effect of laminin α4-FRCs on T cell activation and differentiation (Figure 5J). Strikingly, this correlation profile was mirrored in the laminin α5 co-cultures, suggesting an activating effect of laminin α5-FRCs on T cells (Figure 5J).

Overall, these data indicate that the relative abundance of T cell subsets, in the absence of any external stimulus, is affected by the microenvironment created by the FRCs, which are in turn influenced by the ECM that they are in contact with. Consistent with the tolerogenic or pro-inflammatory transcriptional profiles of either laminin α4-FRC or laminin α5-FRC, respectively, laminin α4-FRCs co-cultures led to tissue-resident T cells with decreased proliferation of effector cells, while laminin α5-FRC co-cultures induced a decrease in the naiïve T cell population accompanied by increased proliferation, especially of effector T cells.

## Discussion

The human LN ECM is composed of various types of ECM proteins, of which collagen is the most abundant ECM protein.^9^ Human LN ECM is the most physiologically relevant coating for FRC culture *in vitro*. Since entire human LNs are used to obtain human LN ECM, which is sparsely available, we sought alternatives to properly grow human FRCs *in vitro* for use in tissue cultures. Here, we demonstrate that, although not functionally assessed, human FRCs cultured on collagen transcriptionally resemble FRCs cultured on native human lymph node (LN) ECM, indicating that collagen is a suitable substitute for human LN ECM as a coating for *in vitro* FRC culture. Besides the structural function of ECM within LNs, some ECM proteins also directly affect immune cells.^21^^,^^22^^,^^24^ Especially two types of laminins, laminin α4 and laminin α5, are associated with either tolerogenic or activating functions on lymphocytes in LNs.^21^^,^^22^^,^^24^ In this study, we show that, depending on the type of laminin, the transcriptional profiles of FRCs differ in their capacity to control the activation status of the immune system in the absence of any immune receptor trigger. While laminin α4 induced a tolerogenic transcriptional profile in human FRCs with increased TGFβ signaling, laminin α5-FRCs demonstrated a pro-inflammatory transcriptional profile based on increased pro-inflammatory cytokine and chemokine transcript counts. We were able to functionally address whether these differences affect the T cells that FRCs interact with by using autologous human LN FRC-T cell co-cultures in the absence of an external stimulus. Laminin α4-FRCs promoted a tissue-resident T cell subset. On the other hand, co-culture of laminin α5-FRCs with T cells resulted in decreased naive T cell numbers, while T cell proliferation was increased, especially in effector T cells.

Previous studies highlight the importance of laminins in the immunological state of mouse LNs.^21^^,^^22^^,^^24^^,^^25^ In activated mouse LNs laminin α5 is the predominant laminin which stimulated mouse and human CD4^+^ T cell activation, proliferation and differentiation toward effector T cells, including Th1, Th2 and Th17 cells.^21^ On the other hand, in resting mouse LN, laminin α4 is mainly present which directly suppressed CD4^+^ T cell activation and proliferation, and promoted Treg polarization.^21^ Recently, Song et al.^26^ demonstrated in mouse models that a unique laminin α5 isoform, laminin 523, affects the follicle-bordering FRC phenotype, influencing T cell-dependent antibody immune responses. The authors hypothesize that the conduit system basement membrane components act as a type of tissue memory that instructs the differentiation of specialized FRC subsets, required for the normal functioning of LNs.^26^ Our findings support this hypothesis, showing that, besides the direct roles of laminins on T cells, human FRCs are also directly instructed by the ECM, which in turn can affect T cells. Here, we have identified differentially expressed immunomodulatory-related gene set clusters in FRCs grown on laminin α4 or laminin α5. The gene set clusters related to immune activation were enriched in laminin α5*: i.e*., immune response and T cell proliferation; and immune processes involving mast and myeloid cells. Conversely, gene set clusters related to immune homeostasis were enriched in laminin α4: *i.e*., development of various tissues; cytoskeletal dynamics; and TGFβ signaling. Human FRCs have previously been described to dampen the proliferation of peripheral blood mononuclear cells (PBMC)- derived T cells during anti-CD3/CD28/CD2 activation using 4 pathways namely, indoleamine-2,3-dioxygenase, adenosine 2A Receptor, prostaglandin E2, and transforming growth factor β receptor (TGFβR).^11^ In our FRC bulk RNAseq data, we only found one of these 4 pathways to be differentially expressed, namely an increase of *TGFB3* transcripts in laminin α4-cultured FRCs. However, *Knoblich* et al.^11^ found these pathways through which FRCs control T cell proliferation and differentiation in human FRC co-cultures with allogeneic PBMC T cells under anti-CD3/CD28/CD2 stimulation, suggesting that activation signals or T cell contact may stimulate FRCs to produce these factors. Recently, another pathway has been described for both mouse and human FRCs to dampen and control T cell responses.^20^^,^^52^ HLA-DR-expressing FRCs are crucial in maintaining peripheral tolerance within LNs by the maintenance and induction of Tregs.^20^^,^^52^ Our analysis of human FRCs cultured on different ECM coatings shows that laminin α4-cultured FRCs have the highest percentage of HLA-DR on their cell surface, which can contribute to the suggested tolerogenic profile of laminin α4 FRCs. The question remains how ECM-derived signals are integrated by FRCs. We have shown that the receptor for laminin α4, CD146, co-localizes with laminin α4 in human LNs. In addition to CD146, multiple integrins have been found to bind to laminins (including α1β1, α2β1, α3β1, α6β1, α10β1, α6β4, α7β1, and αvβ3).^59^ Finally, FRCs are shown to express syndecans, which can bind to ECM components.^13^^,^^59^ However, future studies are required to study the exact molecular mechanism underlying the sensing and subsequent response of FRCs to ECM.

While the initial observations of the differences in T cell responses to different laminins were done using mouse models, we here now demonstrate that the presence of different forms of laminins in the LN ECM composition influences the immune status of T cells in human LNs as well. Since T cells are more likely to receive signals from the LN ECM indirectly via FRCs, which enwrap the LN ECM, we performed autologous FRC-T cell co-cultures on the different ECM coats in the absence of any external stimuli to recapitulate the homeostatic human LN microenvironment. In this way, we are able to study the triadic mode of communication between ECM-imprinted FRCs and T cells in an *in vitro* setting. We demonstrated that FRCs, despite their immune-controlling character, significantly decrease naive CD4^+^ T cells, especially in laminin α5 co-cultures. This could be caused by a combination of factors produced by FRCs that regulate T cell survival and proliferation,^60^^,^^61^ which may be more favorable for other T cell subsets rather than naive T cells. Furthermore, in line with previous studies,^20^^,^^52^ we found an increase in the percentage of Tregs in our laminin α4 and laminin α5 co-cultures enriched with FRCs. Importantly, we found that the FRCs with the highest percentage of HLA-DR on their cell surface, namely laminin α4-cultured FRCs, induce the highest percentage of Tregs. Functional Tregs are required to suppress CD4^+^ effector and memory T cell proliferation and activation in order to control an immune response.^56^^,^^57^^,^^58^ Although we did not perform Treg suppression assays,^56^^,^^57^^,^^58^ the appearance of Ki67^+^ T_EFF_ cells in the laminin α5 co-cultures suggests that the Tregs induced by laminin α5-cultured FRCs were not functionally able to suppress T_EFF_ cell proliferation. Conversely, laminin α4-cultured FRCs might be able to inhibit T_EFF_ cell proliferation, as seen by the unaltered fold change in Ki67^+^ T_EFF_ cells. This is in line with studies in mice in which ablation of FRC-laminin α4 resulted in T cell differentiation into T effector cells, inducing a relatively pro-immune LN environment similar to the laminin α5 co-cultures,^23^ highlighting the importance of laminin α4 in promoting a tolerogenic LN environment.^21^ The laminin α4-FRCs were also able to induce a significant increase in CD69 on T cells. While CD69 is an early activation marker, it is also a putative tissue-residency marker, as its expression inhibits expression of S1PR1, necessary for T cells to respond to the migratory cue S1P.^62^^,^^63^ However, lymphoid tissue residency is complex, and other markers such as CCR7, CD103, and CD62L might also be involved.^64^^,^^65^^,^^66^^,^^67^ Yet, based on CD69 T cell expression, laminin α4-FRCs seem to promote a tissue-resident CD4^+^ T cell phenotype in human LNs. Further study is required to identify which FRC-derived signals control T cell behavior in response to the ECM.

The tolerogenic transcriptional profile of laminin α4-FRCs is further supported by the identification of gene set clusters associated with metabolism in our FRC bulk RNA-seq data. While lipid metabolism was enriched in collagen-FRCs and amino acid metabolism was enriched in laminin α5-FRCs, glycolysis was enriched in laminin α4-FRCs. FRCs have previously been described to undergo metabolic reprogramming during tolerance and immunity.^68^ In a tolerogenic, immune suppressive environment such as cancer, enhanced glycolytic activity in human FRCs has been detected in head/neck tumor-and melanoma-draining LNs, promoting T cell exhaustion and relaxation of the FRC cytoskeleton to aid tumor metastasis. This resulted in diminished anti-tumor immunity.^68^^,^^69^^,^^70^^,^^71^ Furthermore, tumor cells also produce laminin α4 during tumor progression, which stimulates tumor cell migration via binding to CD146.^50^ In mice, laminin α4 is linked to a tolerogenic LN environment with increased levels of Tregs and lowered CD4^+^ T cell activation.^21^^,^^22^^,^^23^^,^^24^ Our observation that glycolysis is enhanced in laminin α4-cultured FRCs supports the hypothesis that laminin α4-cultured FRCs contribute to an immune-inhibitory environment. However, the effect of metabolic reprogramming on FRC cellular functions is context-dependent, as glycolysis is also increased in murine FRCs under inflammatory conditions by IL-17,^68^^,^^72^ warranting further investigation.

LN ECM is produced by FRCs and used to form a reticular conduit system to transport incoming fluids containing soluble factors to which a possible adaptive immune response can be initiated.^7^ In mice, the FRC-specific loss of laminin α4 resulted in impaired conduit integrity and fewer and more disorganized collagen fibers.^23^ Our bulk RNAseq data of human FRCs grown on different ECM coatings supports these findings as we show that human FRCs grown on laminin α4 have increased transcript levels of various ECM genes, including *COL1A2* and *COL4A1* (collagen type 1 and 4), *FN* (fibronectin) and *TNC* (tenascin C), as compared to laminin α5-cultured FRCs. Besides collagen and laminins, human LNs contain many other ECM molecules such as vitronectin, decorin and lumican,^7^^,^^9^ which could as well have specific, unknown roles in the functioning of FRCs.^7^ Human LN ECM composition is thus more complex than only one ECM molecule, and the ratio in which the ECM molecules are present may determine FRC and, indirectly, T cell functionality. Future studies should attempt to combine single-protein ECM molecules such as laminins and basement membrane collagen type IV,^7^ to better understand the effect of the complex native LN ECM on FRCs and T cells. Recently, we have developed a 3D LN model using collagen type 1 hydrogels to study human immune responses in a more physiologically relevant setting.^44^^,^^73^ In order to optimize such 3D LN models, attempts should be made to integrate other ECM molecules, such as laminins, to allow an LN microenvironment that better recapitulates the *in vivo* human LN.

Finally, LN ECM is also a dynamic structure, which is remodeled and changed in protein composition depending on the activation state of a LN.^7^^,^^74^ Such changes in LN ECM composition could possibly become permanent in chronic inflammatory conditions, resulting in the inability to control lymphocyte proliferation and differentiation. Future studies are warranted to investigate the complex interaction of LN ECM, FRCs, and immune cells during health and disease.

In summary, we here reveal a new paradigm in which ECM not only provides structural support to an LN, but also affects the immunomodulatory properties of FRCs and subsequently the immune status of CD4^+^ T cells, steering the environment in which adaptive immune responses are initiated. Studying the complex crosstalk between LN ECM, FRCs, and immune cells will allow a better understanding of human immune responses.

### Limitations of the study

Human LN material is heterogeneous and therefore donor variation is present in this study. We did not assess the potential influence of sex on the reported outcomes. Furthermore, the LN ECM is complex in its structure and composition, making it challenging to study direct functional effects on FRCs and the molecular pathways underlying the interaction of ECM with its receptors on FRCs *in vitro*. Although FRCs cultured on native LN ECM transcriptionally resembled FRCs cultured on collagen type I, the functional properties of these native LN ECM-cultured FRCs were not assessed, and it therefore remains unclear to what extent transcriptional similarity translates into equivalent functional behavior. By using a reductionistic 2D co-culture system, we could dissect the effect of one single ECM component on FRCs and indirectly on T cells. However, with this system, we cannot fully functionally assess the effect of the more complex *in vivo* LN ECM, nor could we control for the ECM produced by the FRCs in the co-culture, which may contribute to the observed T cell responses. Moreover, as we cannot ascertain that the cultured FRCs recapitulate the full spectrum of *in vivo* FRC subsets, our T cell co-culture findings should be interpreted primarily as mechanistic rather than a direct reflection of the *in vivo* LN setting. Finally, the pro-inflammatory and tolerogenic FRC profiles described here are mainly based on transcriptional signatures, while effector T cell and Treg suppressive function were not directly tested, leaving the precise functional implications of these transcriptional states to be further explored.

## Resource availability

### Lead contact

Further information and requests for resources and reagents should be directed to and will be fulfilled by the lead contact: Prof. Dr. Reina E. Mebius (r.mebius@amsterdamumc.nl).

### Materials availability

This study did not generate new unique reagents.

### Data and code availability

• Bulk RNA-seq data have been deposited and are publicly available in the SRA database under the BioProject accession number SRA: PRJNA1419556. Reused publicly available datasets are accessible at Zenodo: 14011548^30^ and at GEO: GSE294959.^43^
• This paper does not report original code.
• The additional data that support the findings of this study are available from the corresponding author on reasonable request.

## Acknowledgments

The authors would like to thank and acknowledge the help of the Microscopy & Cytometry Core Facility at Amsterdam UMC (location Vrije Universiteit Amsterdam). We would like to thank Kimberley Ober and Dr. Monique Verstegen from the Erasmus MC, Rotterdam, for sample logistics, and Dr. Jorke Willemse and Marije van Beek from the Erasmus MC, Rotterdam, for the help with LN ECM isolation. We would also like to thank Dr. Alberta G.A. Paul (Liesbeth) for helping with the design of the T cell flow cytometry panels. D.P. is supported by the NWO LymphChip consortium (1292.19.019). J.E.G.R. is supported by a Gravitation 2013 BO grant financed by the Dutch Research Council (NWO) as part of the Institute for Chemical Immunology (ICI; 024.002.009). L.J.W.v.d.L. is supported by funding from the Convergence Health Technology Flagship grant (Organ Transplantation). C.M.d.W. is supported by the Cancer Center Amsterdam (grants CCA2019-9-57 and CCA2020-9-73) and the Dutch Cancer Foundation (KWF; young investigator grant 2022-4 EXPL/14641). Figures 1A, 4A, and 5A are created using BioRender.com.

## Author contributions

Conceptualization, D.P., J.E.G.R, C.M.W., and R.E.M; methodology, D.P., J.E.G.R, T.K., C.M.W., and R.E.M; software, D.P., J.E.G.R., and M.K.; validation, D.P. and J.E.G.R.; formal analysis, D.P., J.E.G.R., and M.K.; investigation, D.P. and J.E.G.R; resources, H.P.R. and L.J.W.L.; data curation, D.P. and J.E.G.R.; writing – original draft, D.P. and J.E.G.R.; writing-review and editing, D.P., J.E.G.R., C.M.W., and R.E.M.; visualization, D.P. and J.E.G.R.; supervision, C.M.W. and R.E.M.; funding acquisition, R.E.M.

## Declaration of interests

The authors declare no competing interests.

## STAR★Methods

### Key resources table

**Table:** 

REAGENT or RESOURCE	SOURCE	IDENTIFIER
**Antibodies**		

Anti-HLA-DR - PE	Biolegend	Cat# 307606; RRID: AB_314684
Anti-CD34 – Pe-Cy7	Biolegend	Cat# 343516; RRID: AB_1877251
Anti-CD31 – AF488	Biolegend	Cat# 303110; RRID: AB_493074
Anti-BST1/CD157 - FITC	Sino Biological	Cat# 10060-MM10
Anti-CD90 – BV785	BioLegend	Cat# 328142; RRID: AB_2734318
Anti-PDPN – AF647	BioLegend	Cat# 337008; RRID: AB_2162063
Anti-CD31 – BV605	BioLegend	Cat# 303122; RRID: AB_2562149
Anti-FAP – AF488	Bio techne ltd	Cat# FAB3715G; RRID: AB_3233893
Anti-PD-L1 – Pe	BioLegend	Cat# 329706; RRID: AB_940368
Anti-CD80 – BUV737	BD Biosciences	Cat# 751732; RRID: AB_2875714
Anti-CD86 – BV650	BioLegend	Cat# 305428; RRID: AB_2563823
Anti-CD45 – eFluor450	eBioscience	Cat# 48-0459-42; RRID: AB_2016677
Anti-HLA-DR – APC-Fire810	BioLegend	Cat# 307673; RRID: AB_2876603
Anti-CD146 – BV711	BioLegend	Cat# 361032; RRID: AB_2800998
Anti-CD11c – cFluor BYG710	Cytek Biosciences	Cat# RC-00181
Anti-CD14 - cFluor V450	Cytek Biosciences	Cat# R7-20003
Anti-CD4 – BUV563	BD Biosciences	Cat# 612912; RRID: AB_2870197
Anti-CD45 – BV570	BioLegend	Cat# 304034; RRID: AB_2563426
Anti-CD3 – Pe-Dazzle594	BioLegend	Cat# 344844; RRID: AB_2616970
Anti-CD8 – cFluor V610	Cytek Biosciences	Cat# R7-20241
Anti-CD19 – cFluor R685	Cytek Biosciences	Cat# R7-20117
Anti-CCR7 – BV421	BioLegend	Cat# 353208; RRID: AB_11203894
Anti-CD62L – AF488	BioLegend	Cat# 304816; RRID: AB_528857
Anti-CD25 – Pe-Cy7	BioLegend	Cat# 356108; RRID: AB_2561975
Anti-CD69 – BUV737	Thermo Fisher Scientific	Cat# 367-0699-42; RRID: AB_2895981
Anti-CD45RA – PerCP	BioLegend	Cat# 304156; RRID: AB_2616997
Anti-PD-1 – BV785	BioLegend	Cat# 329930; RRID: AB_2563443
Anti-CD45RO – BUV805	BD Biosciences	Cat# 748367; RRID: AB_2872786
Anti-CD127 – APC-R700	BD Biosciences	Cat# 565185; RRID: AB_2739099
Anti-CD27 – APC-Fire810	BioLegend	Cat# 302864; RRID: AB_2894450
Anti-HLA-DR – BUV395	BD Biosciences	Cat# 564040; RRID: AB_2738558
Anti-CTLA4 – APC	Cytek Biosciences	Cat# 20-1529
Anti-Ki67 – BV650	BD Biosciences	Cat# 563757; RRID: AB_2688008
Anti-FoxP3 – PE	BioLegend	Cat# 320208; RRID: AB_492982
Anti-CD3	Agilent	Cat# A0452; RRID: AB_2335677
Anti-laminin a4	Origene	Cat# TrueMab TA805721; RRID: AB_2627834
Anti-CD146	LSbio	Cat# LS-C338736
Anti-CD31	Abcam	Cat# ab32457; RRID: AB_726369
Goat-*anti*-mouse – AF647	Thermo Fisher Scientific	Cat# A21240; RRID: AB_2535809
Goat-*anti*-mouse – AF555	Thermo Fisher Scientific	Cat# A21147; RRID: AB_2535783
Goat-*anti*-rabbit – AF488	Thermo Fisher Scientific	Cat# A11008; RRID: AB_143165

**Biological samples**		

Lymph node of donor #1 (male, 78 years)	Erasmus MC, Rotterdam	N/A
Lymph node of donor #2 (male, 50 years)	Erasmus MC, Rotterdam	N/A
Lymph node of donor #3 (female, 39 years)	Erasmus MC, Rotterdam	N/A
Lymph node of donor #4 (male, 53 years)	Erasmus MC, Rotterdam	N/A
Lymph node of donor #5 (female, 48 years)	Erasmus MC, Rotterdam	N/A
Lymph node of donor #6 (male, 71 years)	Erasmus MC, Rotterdam	N/A
Lymph node of donor #7 (female, 53 years)	Erasmus MC, Rotterdam	N/A
Lymph node of donor #8 (male, 28 years)	Erasmus MC, Rotterdam	N/A
Lymph node of donor #9 (female, 48 years)	Erasmus MC, Rotterdam	N/A
Lymph node of donor #10 (female, 70 years)	Erasmus MC, Rotterdam	N/A
Lymph node of donor #11 (male, 66 years)	Erasmus MC, Rotterdam	N/A
Lymph node of donor #12 (male, 65 years)	Erasmus MC, Rotterdam	N/A
Lymph node of donor #13 (female, 65 years)	Erasmus MC, Rotterdam	N/A
Lymph node of donor #14 (female, 27 years)	Erasmus MC, Rotterdam	N/A
Lymph node of donor #15 (female, 73 years)	Erasmus MC, Rotterdam	N/A
Lymph node of donor #16 (female, 20 years)	Erasmus MC, Rotterdam	N/A
Lymph node of donor #17 (female, 67 years)	Erasmus MC, Rotterdam	N/A

**Chemicals, peptides, and recombinant proteins**		

Belzer UW® Cold Storage Solution	Bridge to Life, University of Wisconsin	
DNAse I	Sigma-Aldrich	Cat# 11284932001
Pepsin	Sigma-Aldrich	Cat# P6887
Dispase II	Sigma-Aldrich	Cat# 04942078001
Collagenase P	Sigma-Aldrich	Cat# 11213857001
RPMI 1640 Medium	Gibco	Cat# 11875093
EDTA	Sigma-Aldrich	Cat# 03677
Fetal bovine serum (FBS)	Serana	Cat# S-FBS-CO-015
Dulbecco’s Modified Eagle Medium (DMEM), high glucose	Gibco	Cat# 11965092
Penicillin/Streptomycin/Glutamine	Capricorn Scientific	Cat# PS-B
Insulin/Transferrin/Selenium	Gibco	Cat# 41400045
Bovine Collagen Type 1	Sigma-Aldrich	Cat# C9791
Human recombinant laminin-421	BioLamina	Cat# LN421-0501
Human recombinant laminin-521	BioLamina	Cat# LN521-05
TrypLE Select Enzyme	Gibco	Cat# A1217701
IMDM	Gibco	Cat# 12440053
Fixable Viability Dye eFluor^TM^ 780	Thermo Fisher Scientific	Cat# 65–0865-14
Trizol LS Reagent	Thermo Fisher Scientific	Cat# 10296010
Bovine Serum Albumin (BSA)	Sigma-Aldrich	Cat# 10735094001
Brilliant Stain Buffer	BD Biosciences	Cat# 563794
Paraformaldehyde	Electron Microscopy Sciences	Cat# 157-4
DAPI	Thermo Fisher Scientific	Cat# D1306
Fluoromount-G	SouthernBiotech	Cat# 0100-01

**Critical commercial assays**		

MagniSort^TM^ Human CD4^+^ T cell Enrichment Kit	Thermo Fisher Scientific	Cat# 8804-6811-74
Foxp3/Transcription Factor Staining Buffer Set	Thermo Fisher Scientific	Cat# 00-5523-00
LEGENDplex™ Human Essential Immune Response Panel	Biolegend	Cat# 740930
LEGENDplex™ stromal custom panel 1778	Biolegend	Cat# 90007212

**Deposited data**		

Bulk RNAseq data	This paper	SRA: PRJNA1419556
Single cell RNAseq data	Public dataset: Grasso et al.^30^	Zenodo: 14011548
Single cell RNAseq data	Public dataset: Fergusson et al.^43^	GEO: GSE294959

**Software and algorithms**		

R (version 4.2.1)	The R Foundation	https://www.rproject.org/
OMIQ	Dotmatics	www.omiq.ai
FlowJo^TM^ Software (version 10.10)	BD Biosciences	https://www.flowjo.com
Graphpad Prism 10	GraphPad Software	https://www.graphpad.com
ImageJ	National Institutes of Health	https://imagej.net
LEGENDplex Data Analysis Software Suite	Biolegend	LEGENDplex™ Software

### Experimental model and study participants details

#### Human lymph nodes

Human LNs were collected from donors during liver transplant procedures, performed at the Erasmus MC, Rotterdam, The Netherlands in accordance with the Medical Ethical Committee (Medisch Ethische Toetsings Commissie; METC) of Erasmus MC (ethical approval reference number MEC-2014-060). All patients (liver transplant recipients) gave written informed consent to use their donor tissue. The LNs were resected along the hepatic artery and portal vein in the porta hepatis from the donor liver. Donor age and sex are shown in the key resources table and in Table S5. Further information regarding donor ethnicity, race, ancestry and socioeconomic status has not been documented upon collection of the tissues. LNs were transported in Belzer University of Wisconsin (UW) cold storage solution (Bridge to Life Ltd, London, England, UK) and were either stored at −80 °C and processed at a later stage for ECM isolation or processed within 72 h after surgery to obtain LN cell suspensions. All LN tissue material, *i.e*., FRCs, immune cells and ECM, were derived from different donors resulting in the use of 17 human LN donors spread across all experiments (to reduce donor variation) (Table S5).

#### Primary human LN FRC culture

Culture flasks were pre-coated with 2 μg/cm^2^ human LN ECM (isolated as described above), 2 μg/cm^2^ collagen type 1 from calf skin (Sigma-Aldrich, St. Louis, MO, USA), or 2 μg/cm^2^ of the human recombinant laminins laminin α4 or laminin α5 (LAM421/LAM521, BioLamina, Sundbyberg, Sweden). Human LN cell suspensions were cultured in DMEM supplemented with 10% FBS, 2% PSG and 1% ITS in pre-coated flasks. Lymphocytes were washed away with PBS after three days of culture to allow optimal FRC growth. Floating cells were washed away twice per week during medium refreshments. Upon confluency, FRCs were harvested with 1× TrypLE Select in PBS (Gibco, Grand Island, NY, USA). This first harvest after seeding an LN suspension is considered as passage 0 FRCs. TrypLE was neutralized using culture medium, and FRCs were passaged to a new pre-coated flask or collected for flow cytometry sorting or flow cytometry analysis. Primary human LN FRC cultures were not tested for mycoplasma contamination.

#### Primary human LN CD4^+^ T cell isolation

Stored human LN cell suspensions were thawed, counted and washed in IMDM (Gibco, Grand Island, NY, USA) 10% FBS 2%PSG. Subsequently, cell pellets were resuspended in cell separation buffer containing 3% FBS, 10 mM EDTA in PBS at a concentration of 10 × 10ˆ^6^ cells/100 μL for magnetic cell separation. CD4^+^ T cell isolation was performed using MagniSort^TM^ Human CD4^+^ T cell Enrichment Kit (Invitrogen by ThermoFisher Scientific) as per the manufacturer’s instructions. Following magnetic separation, cells were washed in DMEM containing 10% FBS, 2% PSG and 1% ITS and prepared for subsequent (co)-culture and flow cytometry purity analysis. CD4^+^ T cell purity (Table S4) and phenotype were assessed by spectral flow cytometry prior to co-culture.

#### Primary human LN FRC and T cell co-culture

24 well plates were coated with either 2 μg/cm^2^ collagen type 1 from calf skin (Sigma-Aldrich, St. Louis, MO, USA), or 2 μg/cm^2^ of the human recombinant laminins laminin α4 or laminin α5 (LAM421/LAM521, BioLamina, Sundbyberg, Sweden). After coating the 24 well plate, FRCs that were previously expanded as described above on the corresponding ECM coating were seeded at 20.000 FRCs per well and cultured for 4 days in DMEM supplemented with 10% FBS, 2% PSG and 1% ITS to reach a high confluency for 4 days at 37°C 5% CO_2_. Subsequently, 200.000 CD4^+^ T cells isolated by magnetic separation were added to the seeded FRCs and co-cultured in DMEM containing 10% FBS, 2% PSG and 1% ITS for 4 days at 37°C 5% CO_2_. Culture of either FRCs or CD4^+^ T cells alone on the same types of ECM coating were taken along as controls. After 4 days of co-culture, CD4^+^ T cells and culture supernatant were obtained. Next, FRCs were harvested using 1× TrypLE Select in PBS, as described previously. Both CD4^+^ T cells and FRCs were analyzed by flow cytometry analysis. Culture supernatants were stored at −20°C after centrifugation for soluble factor analysis.

### Method details

#### Human lymph node ECM isolation

Human LN ECM was isolated based on earlier protocols.^9^^,^^27^^,^^28^^,^^29^ In short, 10 LNs were thawed, combined, and traces of blood and debris were removed using the osmotic effect via washing of the LNs for 30 min with dH_2_O, 1 h with 9% NaCl in dH_2_O and 30 min with dH_2_O. Next, LNs were decellularized using a solution of 4% Triton X-100 and 1% NH_3_. This solution was replaced every hour for a total of 10 repeats, including two overnight incubations of approximately 16 h. After washing for 1 h with dH_2_O, the LNs were incubated with a solution of DNase type 1 (2 mg/L, Sigma-Aldrich, St. Louis, MO, USA) in 0.9% NaCl +100 mM CaCl_2_ + 100 mM MgCl_2_ for 3.5 h at 37 °C. Subsequently, the decellularized LNs were washed twice with dH_2_O and stored at −20 °C. To obtain LN ECM powder, the decellularized LNs were thawed, freeze-dried for 72–96 h using a lyophilizer (Zirbus Technology Sublimer 400, ZIRBUS technology GmbH, Germany) and pulverized using a Retsch ZM200 knife mill with a <200 μm sieve. To prepare an LN ECM suspension appropriate for coating plates, the LN ECM powder was solubilized through enzymatic digestion: 10 mg LN ECM powder was digested in 1 mL digestion solution (1 mg/mL Pepsin (Sigma-Aldrich, St. Louis, MO, USA) in 0.5M Acetic Acid) for 48 h at room temperature. Lastly, the LN ECM coating solution was diluted to 1 mg/mL in 20 mM Acetic Acid and stored at −20 °C, until further usage.

#### Enzymatic digestion of human lymph nodes

To obtain a single cell suspension, human LNs were enzymatically digested as described in earlier protocols,^75^^,^^76^ with a modified time of each digestion cycle to 4 × 10 min intervals. In short, LNs were cut in small pieces and digested with an enzyme mixture of 2.4 mg/mL Dispase II, 0.6 mg/mL Collagenase P and 0.3 mg/mL DNase I (all from Sigma-Aldrich, St. Louis, MO, USA)in RPMI-1640 medium Isolated cells were collected after each digestion cycle in ice-cold phosphate-buffered saline (PBS) supplemented with 5 mM EDTA and 2% fetal calf serum (FCS), and spun down at 300 g for 4 min at 4 °C. The cell pellet was re-suspended in Dulbecco’s Modified Eagle Medium (DMEM) (Gibco, Grand Island, NY, USA) supplemented with 10% FCS, 2% Penicillin/Streptomycin/Glutamine (PSG) and 1% Insulin/Transferrin/Selenium (ITS) (Gibco, Grand Island, NY, USA). All cells were filtered through a 100 μm filter after the last digestion cycle. The obtained human LN cell suspension was cultured *in vitro* to grow out FRCs.

#### Flow cytometry sorting of FRCs for bulk RNA sequencing

The harvested FRCs (from donor 1, 2,3) were filtered through a 70 μm filter and stained for Fixable Viability Dye eFluor^TM^ 780 (1/5000,Invitrogen by ThermoFisher Scientific) in PBS. Fc-receptors were blocked with 10% normal human serum in PBS supplemented with 2% FBS (FACS buffer). Next, the cells were incubated with directly labeled antibodies diluted in FACS buffer, including HLA-DR-PE (1/50), CD34-PE-Cy7 (1/100) and CD31-AF488 (1/100). The harvested cells grown on collagen, laminin α4 and laminin α5 are FRCs, while the cells grown on LN ECM are a mix of FRCs and endothelial cells. Therefore, CD31 was included in the antibody panel of the cells grown on LN ECM, to distinguish FRCs (CD31^neg^) from endothelial cells (CD31^+^). FRCs were filtered through a 40 μm filter before they were flow cytometry sorted using the BD FACSAria Fusion (BD Biosciences, NJ, USA) with a 100 μm tip and collected into PBS with 5% FBS. The FRCs were sorted into MHCII^neg^CD34^+^, MHCII^neg^CD34^neg^ or MHCII^+^CD34^neg^ cells, representing immature, intermediate and mature FRCs, respectively.^77^ Due to the lack of MHCII expression on FRCs grown on laminin α5, these samples were only sorted into MHCII^neg^CD34^+^ and MHCII^neg^CD34^neg^ cells (Table S6). Single stains on beads, an unstained sample, and Fluorescence Minus One (FMO) controls were used for compensation and gating determination. Autofluorescence was compensated using the unstained sample and an extra channel containing the highest autofluorescence (Violet E). Per sample, 10.000 sorted cells were aliquoted in FACS buffer and 1:3 diluted in Trizol LS (Invitrogen by ThermoFisher Scientific) and stored at −80 °C. Frozen samples were shipped for RNA extraction and bulk RNAseq by Single Cell Discoveries (Utrecht, Netherlands) with a sequencing depth of 10 million reads per sample. Library preparation was performed according to the Cel-seq2 protocol.^78^

#### Bulk RNA sequencing analysis

Principal Component Analysis (PCA) was performed on all samples using the DESeq2 package in R (version 4.2.1),^79^ to visualize the data, and to assess overall variation across the samples. The variance is mostly induced by the type of coating and not the sorted populations (Figure S3). Therefore, all sorted populations for each donor were included for follow-up analysis to examine the effect of ECM coating, this resulted in 6 samples for either native lymph node ECM, collagen type 1 and laminin α4, and 4 samples for laminin α5. Differential gene expression analysis was performed per coating using the DESeq2 package (version 1.36.0) in R.^79^ Differentially expressed genes (DEGs) between the coatings were defined based on a Wald test adjusted *p*-value <0.05. Gene Set Enrichment Analysis (GSEA) was performed using the gseGO function of the clusterProfiler package (version4.4.4) in R, using the standard *pvalueCutoff* = 0.05.^80^ The significant gene sets were clustered using the GScluster package (version 1.1.10) in R, with the settings GsQCutoff = 0.04 and GQCutoff = 0.05.^81^ Gene set clusters were annotated based on the gene sets within the cluster. To examine the read counts for specific genes of interest across the coatings, the transcript counts were normalized by the estimated size factor, which is a scaling factor based on the comparisons of gene counts to the geometric mean across samples. Variably expressed genes between the coatings were identified by One-way ANOVA analysis with Tukey’s multiple comparisons test and visualized as mean ± standard deviation, using Prism10 software (GraphPad).

#### Comparison FRC bulk RNA sequencing data with publicly available datasets

To validate whether our cultured FRCs represent FRCs in a homeostatic lymph node, we compared our bulk RNAseq with a publicly available scRNAseq dataset of human FRCs from homeostatic lymph nodes (Figure S1).^30^ This integrated dataset containing the data of Grasso et al.*,*^30^ Abe et al.*,*^82^ and Kapoor et al.^4^ was obtained from the publicly available Zenodo at https://zenodo.org/uploads/14011548 (reference number 14011548). This dataset contains 12 clusters, of which cluster 9 was annotated as B cells and cluster 4 as keratinocytes, while the other clusters were annotated as stromal cells.^30^ From this integrated scRNAseq dataset the top-ten differentially expressed genes per cluster were determined using the FindAllMarkers function of the R Seurat Package (v5.3.1). Two heatmaps were made with the resulting gene list; one of the integrated scRNAseq dataset containing the data of of Grasso et al.*,*^30^ Abe et al.*,*^82^ and Kapoor et al.^4^ and one on the log2(counts+0.01) of our bulkRNA seq. These heatmaps are shown side to side in (Figure S1).

To validate our FRC bulk RNAseq results against resting or activated LNs, we again compared our data with another publicly available human LN single-cell RNAseq dataset.^43^ This dataset comprises human LN slice cultures stimulated for 20 h with a vaccine adjuvant (LMQ) alongside unstimulated control LN slices. Processed.h5 data files were obtained from the publicly available Gene Expression Omnibus (GEO; accession code GSE294959) and were converted to Seurat Objects using Seurat Package (version 5.3.1) in R (version 4.5.0). All donor samples were initially analyzed independently using the standard Seurat “Guided Clustering Tutorial Vignette”(found at webpage https://satijalab.org/seurat/articles/pbmc3k_tutorial)), and subsequently integrated using the “Introduction to scRNA-seq integration” pipeline (found at webpage https://satijalab.org/seurat/archive/v4.3/integration_introduction)). Among the available integration algorithms, Harmony was selected because it produced integrated data that most closely reflected the expected biology. Clustering was performed using default resolution and cluster annotation was conducted by examining the expression of canonical marker genes across clusters, using the same markers reported in Figure 1 of Fergusson et al*.*^43^ To focus specifically on stromal and endothelial cells, the integrated dataset was subset to Cluster 16, which was characterized by the expression of *PECAM1* and *COL1A2*. DEG analysis was then performed only between control and activated LN slice samples from the stromal/endothelial cell cluster. Genes identified as significantly differentially expressed between control and activated LN slices were subsequently crosschecked with differentially expressed genes identified in our FRC bulk RNAseq dataset across ECM coating conditions. Only genes that were significantly differentially expressed in both datasets are visualized in Table S3.

#### Flow cytometry analysis

##### Extracellular flow cytometry staining of human LN FRCs and T cells

Cells were stained for multispectral flow cytometry analysis. Washes were performed by centrifugated at 300-400g for 4 min at 4°C. Cells were first washed with FACS buffer (PBS containing 0.1% bovine serum albumin (BSA; Roche Diagnostics GmbH, Rotkreuz, Switzerland), followed by PBS and subsequently stained with a fixable viability dye eFluor™ 780 (1/4000, Invitrogen by ThermoFisher Scientific) for 10 min. Next, cells were washed two times with FACS buffer and Fc-receptors were blocked for 10 min using blocking buffer containing 10% normal human serum in FACS buffer 1:1 mixed with Brilliant Stain Buffer (BSB) (BD Biosciences, NJ, USA). Without washing, cells were incubated with directly-labelled antibodies detecting extracellular antigens diluted in blocking buffer. For FRC panel 1, the following antibodies were included: BST1-FITC (1/200), CD90-BV785 (1/100), CD34-Pe-Cy7 (1/100), PDPN-AF647 (1/50), HLA-DR-PE (1/100) and CD31-BV605 (1/100). For the FRC panel after FRC -T-cell co-culture (panel 2), the following antibodies were included: FAP-AF488 (1/25), PD-L1-PE (1/25), PDPN-AF647 (1/50), CD80-BUV737 (1/100), CD86-BV650 (1/100), CD45-eFluor450 (1/100), HLA-DR-APC-Fire810 (1/100) and CD146-BV711 (1/100). The T cell purity panel included: CD11c-cFluor BYG710 (1/20), CD14-cFluor V450 (1/20), CD4-BUV563 (1/20), CD45-BV570 (1/50), CD3-PE-Dazzle594 (1/50), CD8-cFluor V610 (1/50) and CD19-cFluor R685 (1/100). Finally, for the T cell functional panel, the following antibodies were included: CD19-cFluor R685 (1/100), CCR-BV421 (1/20), CD62L-AF488 (1/50), CD25-PE-Cy7 (1/20), CD69-PerCP (1/20), CD45ra-PerCP (1/25), CD4-BUV563 (1/20), CD45-BV570 (1/50), PD1-BV785 (1/50), CD3-PE-Dazzle594 (1/50), CD45ro-BUV805 (1/50), CD127-APC-R700 (1/50), CD27-APC-Fire810 (1/50) and HLA-DR-BUV395 (1/100). After 20 min of incubation, cells were washed once with FACS buffer followed by a PBS wash prior to fixation using 2% paraformaldehyde (PFA) (Electron Microscopy Sciences, PA, USA) for 15 min. Subsequently, cells were washed and stored in FACS buffer at 4°C in the dark until acquisition. Samples, fluorescence minus ones (FMOs) and single stains on beads were acquired on an Aurora 5-laser Flow Cytometer (Cytek, Amsterdam, The Netherlands). Autofluorescence (AF) correction of cells was performed as previously described.^83^ Data analysis was conducted using OMIQ software (Dotmatics) and FlowJo software v10 (BD Life Sciences, NJ, USA). A representative gating strategy can be found in Figures S4 and S6.

#### Intracellular flow cytometry staining of human LN T cells

For intracellular staining, T cells were first stained extracellularly as described above. After extracellular antibody incubation, cells were washed once with FACS buffer followed by a PBS wash. Cells were intracellularly stained using the eBioscience™ Foxp3/Transcription Factor Staining Buffer Set (Invitrogen by ThermoFisher Scientific). In short, cells were fixed and permeabilized with Intracellular Fixation and Permeabilization Buffer for 45 min at 4°C in the dark. Subsequently, cells were washed once using the 1× permeabilization buffer diluted in Milli-Q water and stained with the directly-labeled antibodies for intracellular antigens diluted in 1× permeabilization buffer at 4°C in the dark, including: CTLA-4-APC (1/50), Ki67-BV650 (1/100) and FoxP3-PE (1/50).After 60 min, cells were washed two times with FACS buffer and stored in FACS buffer at 4°C in the dark until acquisition. Samples, fluorescence minus ones (FMOs) and single stains on beads were acquired on an Aurora 5-laser Flow Cytometer (Cytek, Amsterdam, The Netherlands). Autofluorescence (AF) correction of cells was performed as previously described.^83^ Data analysis was conducted using OMIQ software (Dotmatics) and FlowJo software v10 (BD Life Sciences, NJ, USA).

#### Cytokine/chemokine detection in culture supernatants

Culture supernatants were analyzed for the presence of cytokines and chemokine using cytokine bead arrays (Human Essential Immune Response LEGENDplex panel and custom stromal LEGENDplex panel 1778, BioLegend, San Diego, CA, USA), according to manufacturer’s instructions. Acquisition was performed on the Attune™ CytPix™ Flow Cytometer (ThermoFisher, Waltham, MA, USA) and protein concentrations were determined using the LEGENDplex Data Analysis Software Suite (BioLegend, San Diego, CA, USA).

#### Immunofluorescence staining of human LNs

For paraffin embedding, healthy human LNs were dehydrated, embedded in paraffin and cut at 5 μm sections for immunofluorescent analysis. Sections were deparaffinized and immersed in Tris-EDTA buffer (pH 9.0) for 5 min at 120°C for antigen retrieval, followed by slowly cooling to room temperature. Sections were then washed with PBS and blocked for 15 min with 1% bovine serum albumin and 10% normal goat serum in PBS. Next, LN sections were stained with primary antibodies, including rabbit anti-human CD3 (1/50), mouse anti-human laminin α4 (1/200), mouse anti-human CD146 (1/200) and rabbit anti-human CD31 (1/200), for 1 h, washed three times with PBS for 2 min, and stained with secondary antibodies for 30 min at room temperature, including goat anti-mouse AF647 (1/400), goat anti-mouse AF555 (1/400) and goat anti-rabbit AF488 (1/400). After staining, LN sections were washed three times with PBS for 2 min and stained with DAPI (5 μg/mL, Invitrogen via ThermoFisher Scientific) for 5 min. After three washes with PBS for 2 min, stained sections were mounted with Fluoromount-G® (SouthernBiotech, Birmingham, AL, USA) and stored in the dark at 4°C until acquisition. Confocal images were acquired using a Nikon AXR (Nikon) and analyzed with ImageJ (Fiji).^84^

### Quantification and statistical analysis

Statistical analysis was conducted using either a paired *t* test, one-way ANOVA or two-way ANOVA with Tukey’s multiple comparisons test in GraphPad Prism 10 software (GraphPad Software Inc., San Diego, CA, USA). For correlation analysis, the base R cor() function and the corrplot() function of the corrplot package (version 0.95) were used in R (version 4.3.2). Differential gene expression was analyzed in R (version 4.3.2) via DESeq2 with Wald test for bulk RNAseq or via the FindAllMarkers function of the R Seurat Package (v5.3.1) for scRNAseq. The statistical tests used are indicated in the figure legends. Differences were considered significant when *p* < 0.05. Independent experimental replicates of the full experiments were not conducted.
