# Supplementary material for: Lymph node extracellular matrix skews the immune function of human fibroblastic reticular cells
Source: iScience. 2026 Sep 16;29(10):117516. doi: 10.1016/j.isci.2026.117516 (PMC13599584; doi:10.1016/j.isci.2026.117516)
Supplement: Supplementary file 1 [file mmc1.pdf]

iScience, Volume *29*

Supplemental information

Lymph node extracellular matrix skews the immune function of human fibroblastic reticular cells

Daphne Panocha, Janna E.G. Roet, Michael de Kok, Tanja Konijn, Henk P. Roest, Luc J.W. van der Laan, Charlotte M. de Winde, and Reina E. Mebius

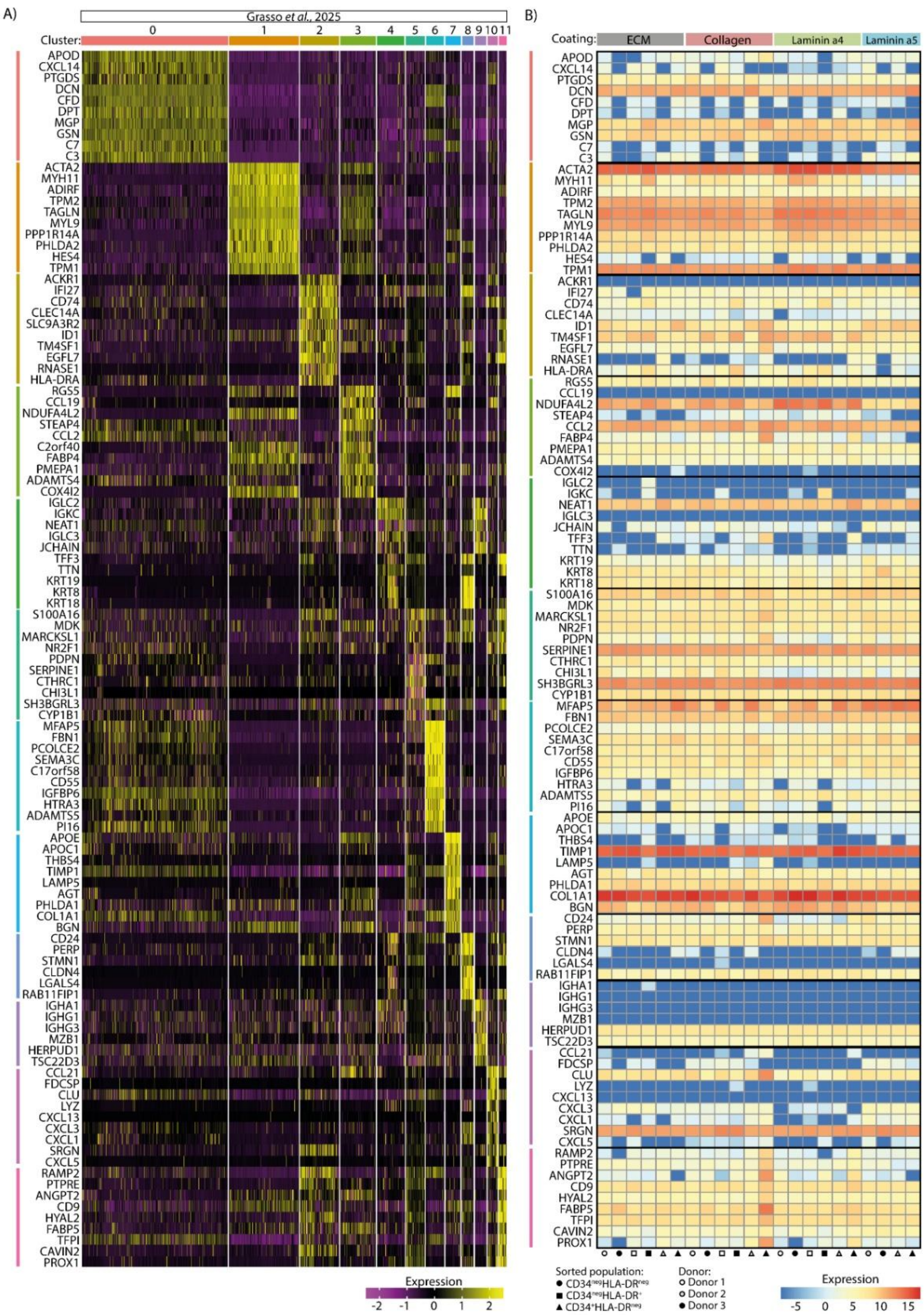

Figure S1. Comparison of bulk RNAseq of cultured FRCs with single cell RNAseq of *ex vivo* FRCs to illustrate partial preservation of stromal transcriptional programs *in vitro*.
Related to Figure 1. A) Heatmap visualizing the top 10 differentially expressed genes per cluster within the publicly available integrated human LN FRC single cell RNA sequencing dataset from Grasso *et al*. [S1]. Clusters 0-7, 10 and 11 were previously annotated as stromal subsets (including multiple FRC-like populations), cluster 9 as B cells and cluster 4 as keratinocytes. B) Heatmap showing gene expressions (Log_2(normalized counts + 0,01)) within the bulk RNA sequencing from the FRCs grown on different coatings: Extracellular matrix (ECM), Collagen type 1, Laminin α4 or Laminin α5 between passage 1-4. The symbols represent the different sorted populations: $CD34^{neg}HLA\text{-}DR^{neg}$ (round), $CD34^{neg}HLA\text{-}DR^{+}$ (square), $CD34^{+}HLA\text{-}DR^{neg}$ (triangle). The filling of the symbols represent the different donors: donor 1 (open), donor 2 (grey-filled), donor 3 (black-filled). Bulk RNAseq data are derived from FRCs between passage 1-4 from donors 1,2 and 3 (ECM: n = 6; collagen: n = 6; laminin α4: n = 6; laminin α5: n = 4; see Table S6).

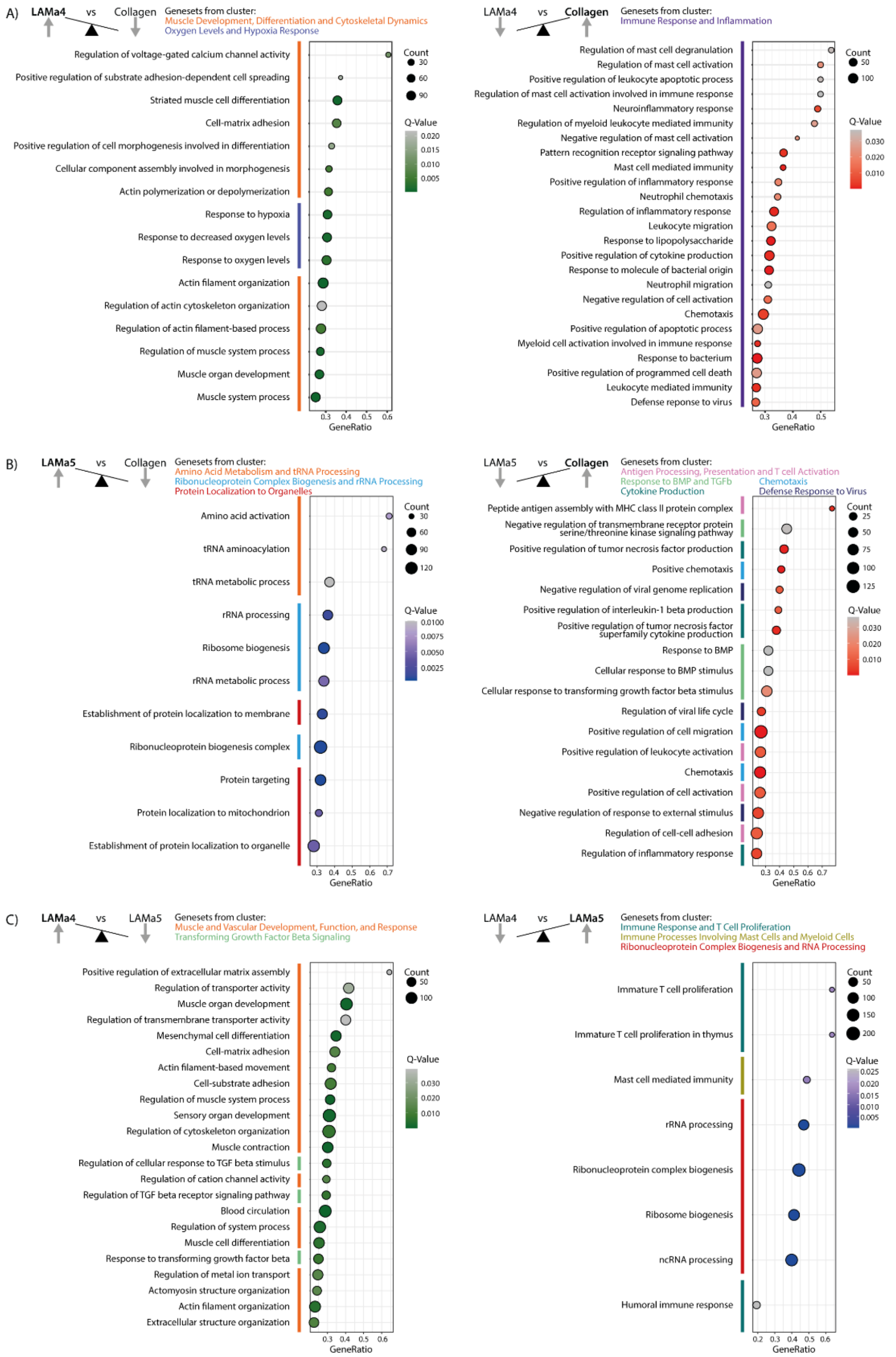

Figure S2. Different gene sets enriched in FRCs grown on collagen, laminin α4 and laminin α5. Related to Figure 2. Dotplots of significantly enriched gene sets from selected clusters identified in FRCs grown on laminin α4 vs collagen (A), laminin α5 vs collagen (B) and laminin α4 vs laminin α5 (C). Per comparison, gene set enrichment analysis was performed based on the differentially expressed genes, followed by gene set cluster analysis. Per selected cluster, the top 4 significant gene sets (based on Q-Value) per subterm are visualized. Dot colour represents statistical significance (Q-Value) and size is proportional to gene count (number of genes annotated in each gene set). GeneRatio: (number of enriched genes) / (number of genes in each gene set). See Table S1 for gene sets per subterm of the selected clusters. Bulk RNAseq data are derived from FRCs between passage 1-4 from donors 1,2 and 3 (collagen: n = 6; laminin α4: n = 6; laminin α5: n = 4). LAMa4/5: laminin α4/5.

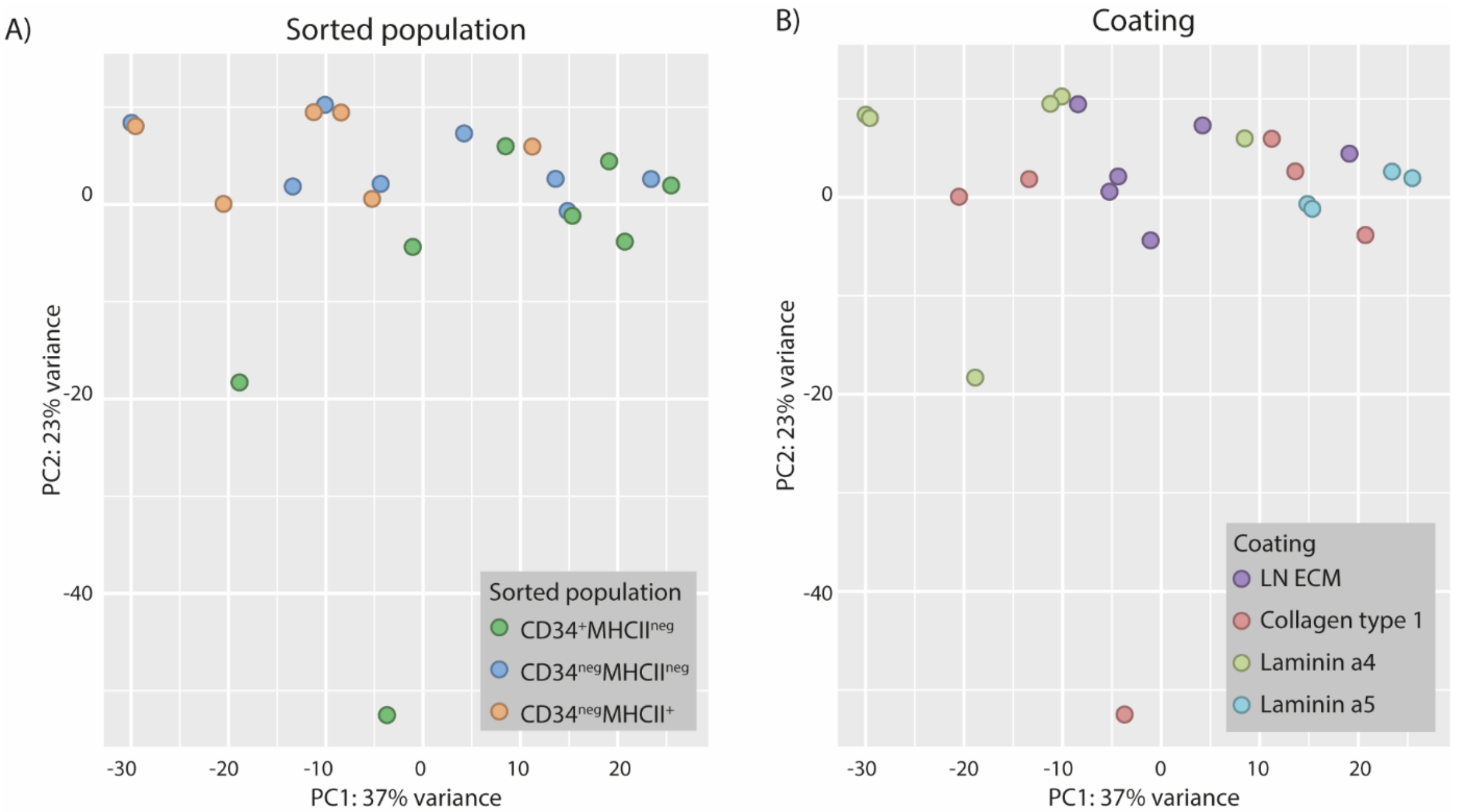

Figure S3. Type of ECM coating induces highest gene expression variance between FRC samples.
Related to STAR methods. Principal component analysis (PCA) plots of bulk RNA sequencing data from FRCs grown on native lymph node ECM, collagen, laminin α4 or laminin α5 and sorted in $CD34^{+}MHCII^{neg}$, $CD34^{neg}MHCII^{neg}$ or $CD34^{neg}MHCII^{+}$ FRCs. The PCA plots visualize all samples based on sorted population (A) or coating (B). Data is from bulk RNAseq samples of FRCs between passage 1-4, derived from donors 1, 2, and 3 ($CD34^{+}MHCII^{neg}$: n = 8; $CD34^{neg}MHCII^{neg}$: n = 8; $CD34^{neg}MHCII^{+}$: n = 6; LN ECM: n = 6; collagen: n = 6; laminin α4: n = 6; laminin α5: n = 4; see Table S6 for sample characteristics). ECM: extracellular matrix, LN: lymph node, MHCII: MHC class II, PC: principal component.

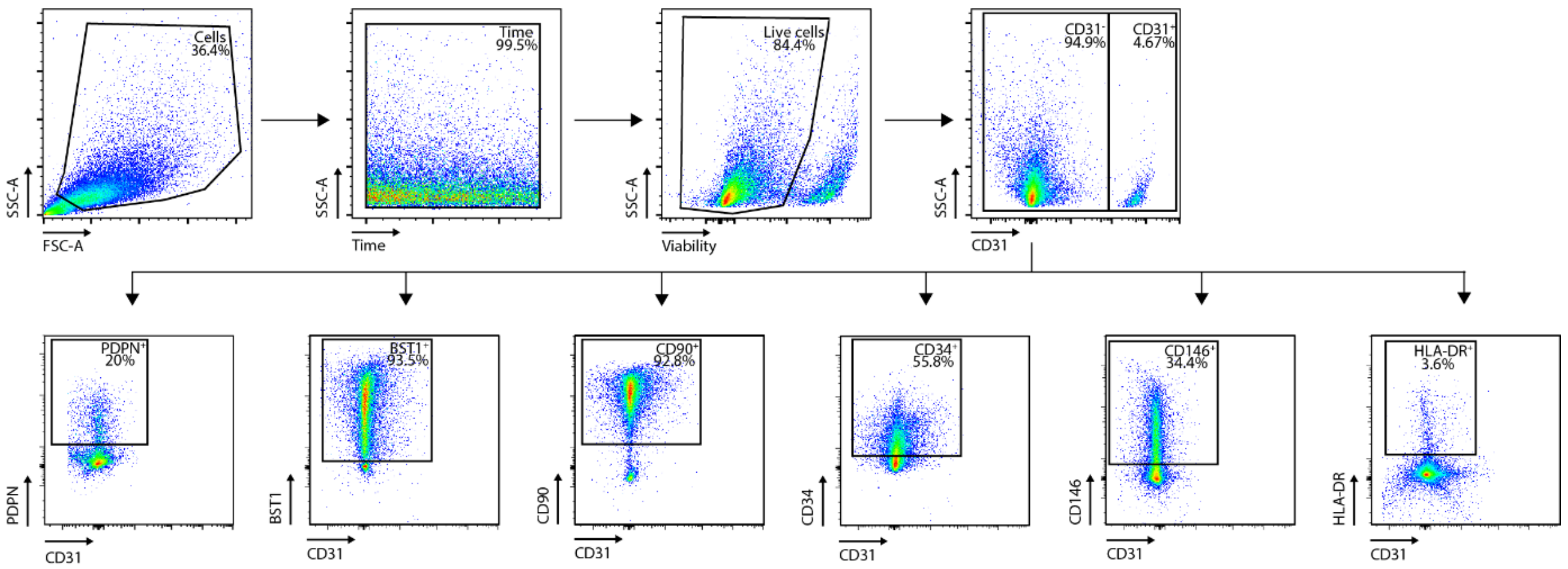

Figure S4. Representative gating strategy for FRC surface markers.
Related to Figure 4. The gating strategy is based on collagen FRCs passage 1, donor 1 and is representative for all four lymph node donors.

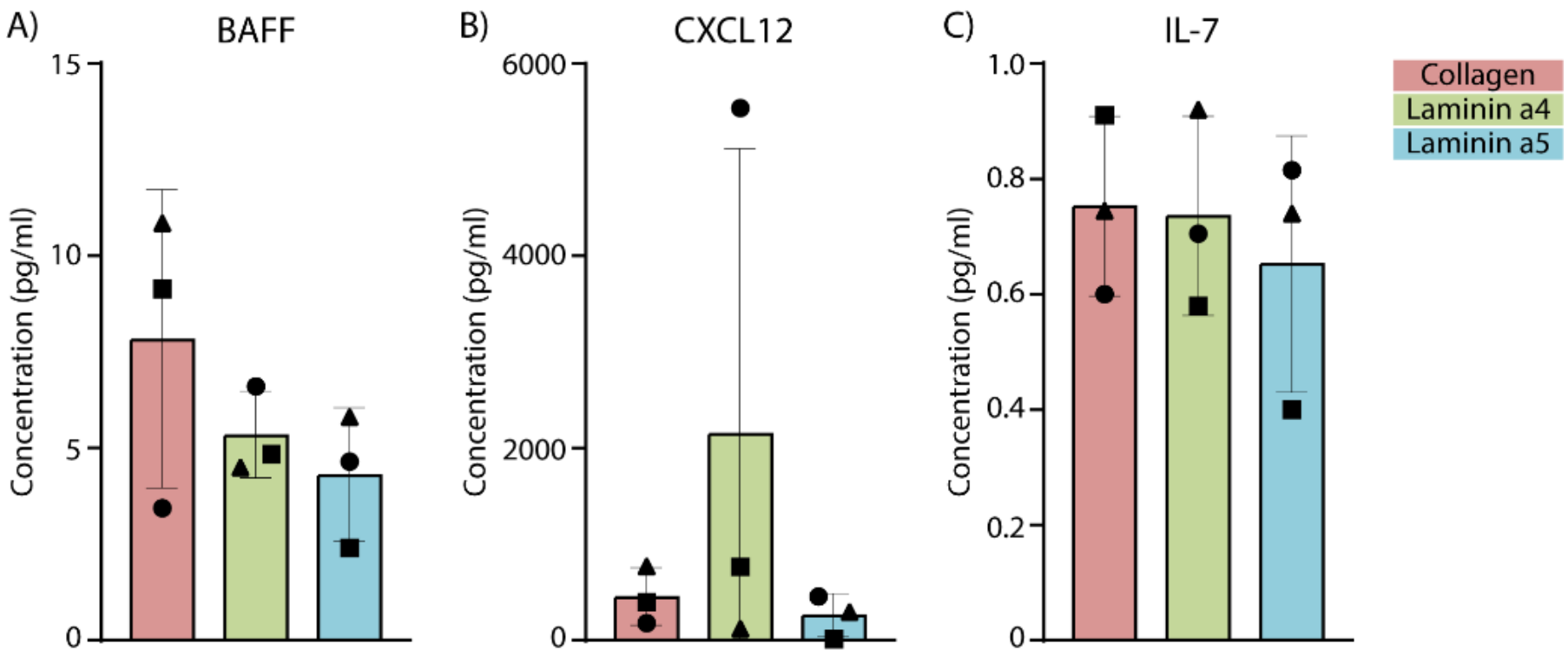

Figure S5. Cytokine and chemokine levels in FRC cultures

Related to Figure 4. Concentration in pg/mL of (A) BAFF, (B) CXCL12, (C) IL-7, in the supernatant of FRCs harvested after 96 hours in culture. APRIL, CCL19, CXCL13, CCL21 could not be detected. Red = collagen; green = laminin α4; blue = laminin α5. N=3 donors. The different symbols represent different donors: square = donor 2, circle = donor 4, triangle = donor 7. Data are represented as mean ± SD.

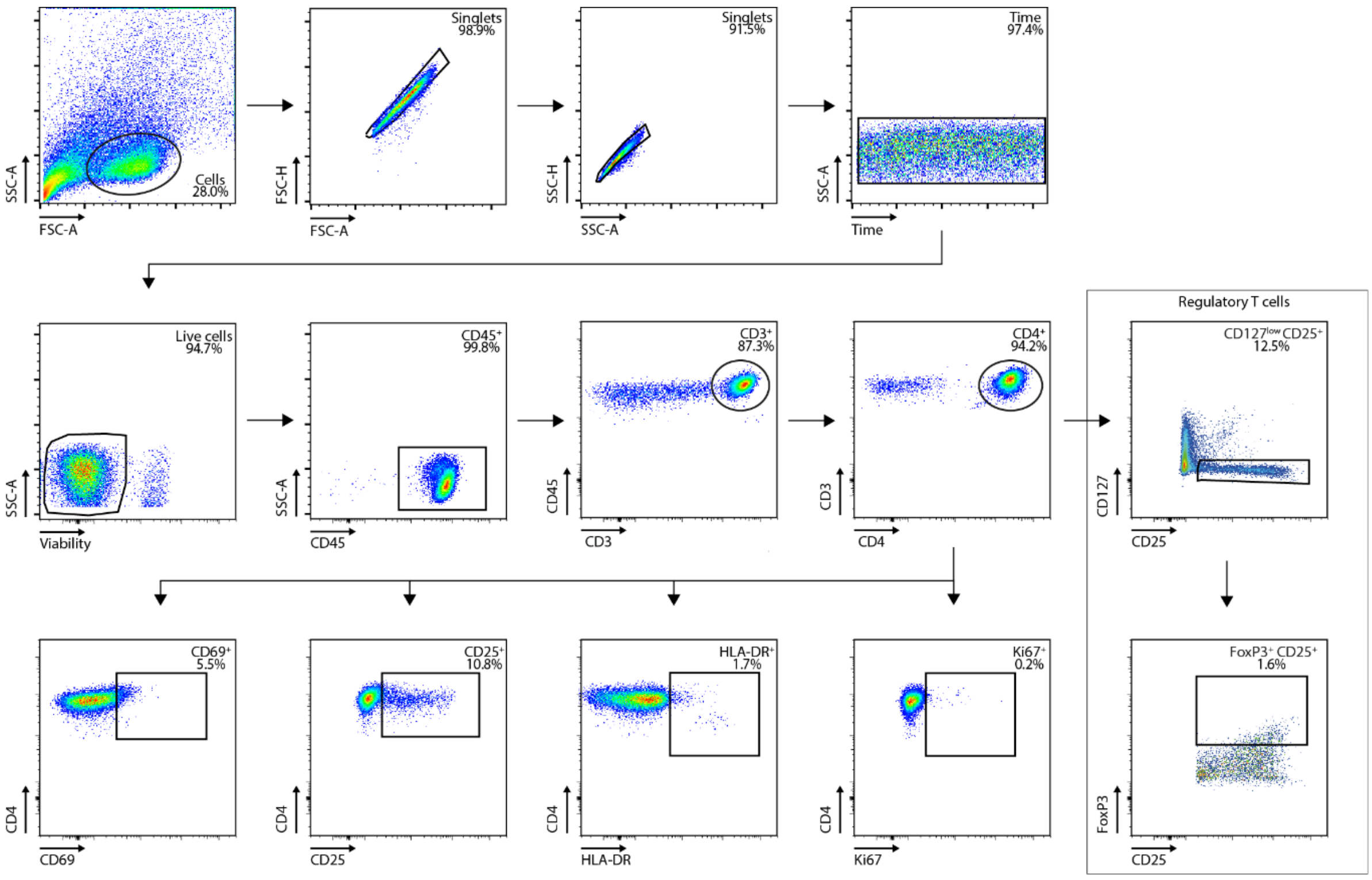

Figure S6. Representative gating strategy for CD4⁺ T cells.

Related to Figure 5. The gating strategy is based on T cells from collagen FRC + T cell co-culture, donor 2 and is representative for all three lymph node donors.

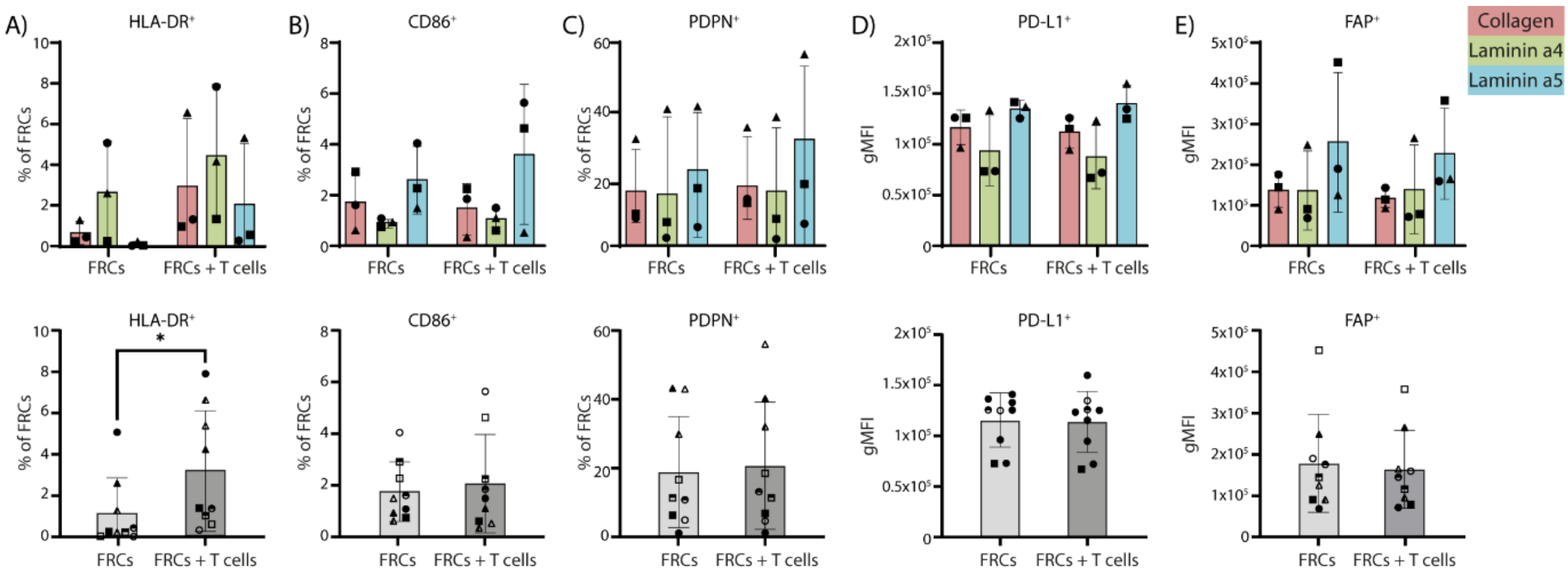

Figure S7. FRC surface markers in co-culture with CD4⁺ T cells.
Related to Figure 5. Percentage of HLA-DR⁺ (A), CD86⁺ (B), PDPN⁺ FRCs (C), and geometric mean fluorescent intensity (gMFI) of PD-L1 within PD-L1⁺ FRCs (D) or gMFI of FAP within FAP⁺ FRCs (E). Top row represents differences per ECM coating; bottom row represents combined data of ECM coatings, 96 hours after co-culture with T cells. Red/half open symbol = collagen; green/closed symbol = laminin α4; blue/open symbol= laminin α5. N=3 donors. Symbol shapes represent different donors: square = donor 2, circle = donor 4, triangle = donor 7. FRCs within the co-culture are at passage 3 (donor 4 and 7) or passage 4 (donor 2). Data are represented as mean ± SD. Paired T test. * = $p < 0,05$.

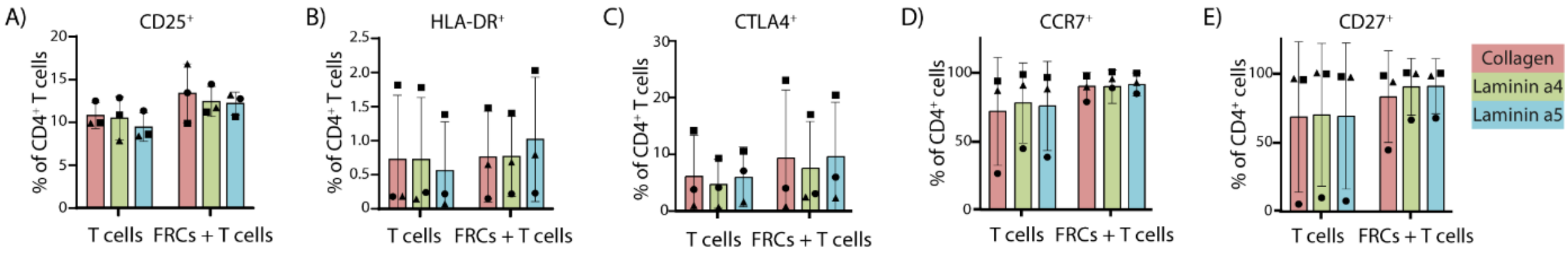

Figure S8. CD4⁺ T cell markers in FRC + T cell co-culture.
Related to Figure 5. Percentage CD25⁺ (A), HLA-DR⁺ (B), CTLA4⁺ (C), CCR7⁺ (D), and CD27⁺ (E) cells of CD4+ T cells. All graphs are 96 hours after co-culture with or without FRCs. Each datapoint represents one donor (mean of technical duplicates); symbol shapes indicate donors: square = donor 2, circle = donor 4, triangle = donor 7. FRCs within the co-culture are at passage 3 (donor 4 and 7) or passage 4 (donor 2). Red = collagen; green = laminin α4; blue = laminin α5. N=3 donors. Data are represented as mean ± SD.

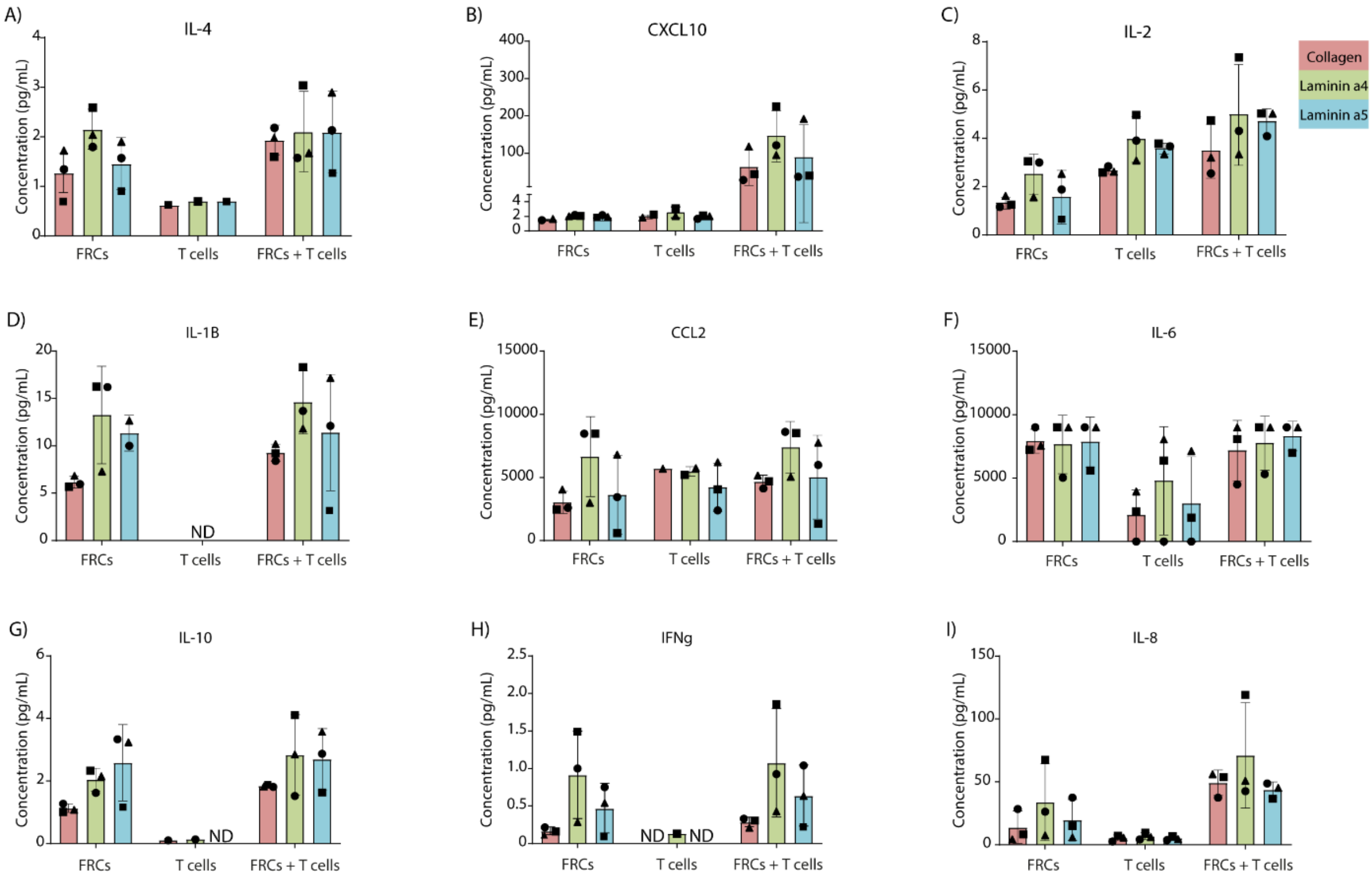

Figure S9. Cytokine and chemokine levels in FRC + T cell co-cultures.
Related to Figure 5. Concentration in pg/mL for FRCs, T cells and FRCs + T cells of (A) IL-4, (B) IL-2, (C) CXCL10, (D) IL-1B, (E) CCL2, (F) IL-6, (G) IL-10, (H) IFNg, and (I) IL-8, 96 hours after (co-)culture. Symbol shapes represent different donors: square = donor 2, circle = donor 4, triangle = donor 7. FRCs within the co-culture are at passage 3 (donor 4 and 7) or passage 4 (donor 2). ND = non-determined. Red = collagen; green = laminin α4; blue = laminin α5. N=3 donors. Data are represented as mean ± SD.

laminin α4 ↑ vs Collagen ↓

Cluster	Subcluster	Gene set	Q-Value
Muscle Development, Differentiation, and Cytoskeletal Dynamics	Muscle System Processes and Development	Muscle system process	0,00001
		Striated muscle cell differentiation	0,00016
		Regulation of muscle system process	0,00102
		Muscle organ development	0,00119
	Cytoskeletal Organization and Actin Dynamics	Actin filament organization	0,00007
		Actin polymerization or depolymerization	0,00529
		Regulation of actin filament-based process	0,00706
		Regulation of actin cytoskeleton organization	0,01972
	Cell Differentiation and Morphogenesis	Cellular component assembly involved in morphogenesis	0,00324
		Cell-matrix adhesion	0,00650
		Positive regulation of cell morphogenesis involved in differentiation	0,00928
		Positive regulation of substrate adhesion-dependent cell spreading	0,01437
		Regulation of voltage-gated calcium channel activity	0,01671
Oxygen Levels and Hypoxia Reponse	X	Response to decreased oxygen levels	0,00371
		Response to hypoxia	0,00376
		Response to oxygen levels	0,00650

laminin α4 ↓ vs Collagen ↑

Cluster	Subcluster	Gene set	Q-Value
Immune Response and Inflammation	Response to Pathogens	Response to bacterium	0,00003
		Response to molecule of bacterial origin	0,00012
		Response to lipopolysaccharide	0,00017
		Defense response to virus	0,00232
	Immune Activation and Regulation	Pattern recognition receptor signaling pathway	0,00055
		Myeloid cell activation involved in immune response	0,00057
		Mast cell mediated immunity	0,00095
		Leukocyte mediated immunity	0,00176
	Inflammatory Response and Regulation	Regulation of inflammatory response	0,00176
		Positive regulation of cytokine production	0,00207
		Positive regulation of inflammatory response	0,00657
		Neuroinflammatory response	0,02238
	Cellular Activation and Apoptosis	Positive regulation of programmed cell death	0,01430
		Positive regulation of apoptotic process	0,01657
		Negative regulation of cell activation	0,01657
		Positive regulation of leukocyte apoptotic process	0,01856
	Chemotaxis and Migration	Chemotaxis	0,00576
		Neutrophil chemotaxis	0,02204
		Leukocyte migration	0,03537
		Neutrophil migration	0,03614
	Mast Cell and Myeloid Regulation	Regulation of mast cell activation	0,02119
		Negative regulation of mast cell activation	0,02119
		Regulation of mast cell activation involved in immune response	0,02574
		Regulation of mast cell degranulation	0,02876
		Regulation of myeloid leukocyte mediated immunity	0,02885

laminin α5 ↑ vs Collagen ↓

Cluster	Subcluster	Gene set	Q-Value
Amino Acid Metabolism and tRNA Processing	X	Amino acid activation	0,00706
		TRNA aminoacylation	0,00840
		TRNA metabolic process	0,01049
Ribonucleoprotein Complex Biogenesis and rRNA Processing	X	Ribosome biogenesis	0,00052
		Ribonucleoprotein complex biogenesis	0,00066
		RRNA processing	0,00067
		RRNA metabolic process	0,00258
Protein Localization to Organelles	X	Protein targeting	0,00011
		Establishment of protein localization to membrane	0,00219
		Establishment of protein localization to organelle	0,00249
		Protein localization to mitochondrion	0,00549

laminin α5 ↓ vs Collagen ↑

Cluster	Subcluster	Gene set	Q-Value
Antigen processing, presentation and T cell activation	X	Peptide antigen assembly with MHC class II protein complex	0,00209
		Positive regulation of leukocyte activation	0,00335
		Regulation of cell-cell adhesion	0,00339
		Positive regulation of cell activation	0,00372
Cytokine production	X	Positive regulation of tumor necrosis factor production	0,00249
		Positive regulation of tumor necrosis factor superfamily cytokine production	0,00385
		Regulation of inflammatory response	0,00706
		Positive regulation of interleukin-1 beta production	0,00706
Defense Response to Virus	X	Negative regulation of response to external stimulus	0,00321
		Regulation of viral life cycle	0,00635
		Negative regulation of viral genome replication	0,00707
Chemotaxis	X	Chemotaxis	0,00009
		Positive chemotaxis	0,00071
		Positive regulation of cell migration	0,00078
Response to BMP and TGFb	X	Negative regulation of transmembrane receptor protein serine/threonine kinase signaling pathway	0,01466
		Cellular response to transforming growth factor beta stimulus	0,01697
		Response to BMP	0,01789
		Cellular response to BMP stimulus	0,01789

laminin α4 ↑ vs laminin α5 ↓

Cluster	Subcluster	Gene set	Q-Value
Transforming Growth Factor Beta Signaling	X	Regulation of transforming growth factor beta receptor signaling pathway	0,00286
		Regulation of cellular response to transforming growth factor beta stimulus	0,00372
		Response to transforming growth factor beta	0,01767
Muscle and Vascular Development, Function, and Response	Muscle System Processes and Development	Muscle contraction	0,00000
		Muscle organ development	0,00026
		Regulation of muscle system process	0,00041
		Muscle cell differentiation	0,00251
	Cytoskeletal Organization and Actin Dynamics	Actin filament organization	0,00518
		Actomyosin structure organization	0,01279
		Actin filament-based movement	0,01698
		Regulation of cytoskeleton organization	0,01959
	Regulation of Transport and Cellular Responses	Regulation of metal ion transport	0,00026
		Regulation of cation channel activity	0,00072
		Regulation of transporter activity	0,00238
		Regulation of transmembrane transporter activity	0,00296
	Tissue and Organ Development	Mesenchymal cell differentiation	0,00017
		Blood circulation	0,00037
		Regulation of system process	0,00043
		Sensory organ development	0,00099
	Extracellular Matrix Organization and Adhesion	Cell-matrix adhesion	0,00778
		Cell-substrate adhesion	0,01142
		Positive regulation of extracellular matrix assembly	0,02374
		Extracellular structure organization	0,02626

laminin α4 ↓ vs laminin α5 ↑

Cluster	Subcluster	Gene set	Q-Value
Immune Processes Involving Mast Cells and Myeloid Cells	X	Mast cell mediated immunity	0,01921
Immune Response and T Cell Proliferation	X	Humoral immune response	0,01921
		Immature T cell proliferation	0,02170
		Immature T cell proliferation in thymus	0,02170
Ribonucleoprotein Complex Biogenesis and RNA Processing	X	Ribonucleoprotein complex biogenesis	0,00000
		NcRNA processing	0,00000
		Ribosome biogenesis	0,00000
		RRNA processing	0,00000

Table S1. Top 4 gene sets within the subclusters of selected gene set clusters in FRCs. The differentially expressed gene sets in FRCs per comparison (laminin α4 vs collagen, laminin α5 vs collagen and laminin α4 vs laminin α5) were determined using gene set enrichment analysis, followed by gene set cluster analysis. The gene sets in selected clusters were manually divided in subclusters. The top 4 gene sets within the subclusters, based on Q-Value, are listed and used in Figure S2. Bulk RNAseq data are derived from FRCs between passage 1-4 from donors 1,2 and 3 (collagen: n = 6; laminin α4: n = 6; laminin α5: n = 4).

Gene	laminin α4 vs Collagen	laminin α5 vs Collagen	laminin α4 vs laminin α5	Additional FRC surface markers
IL7	***			
IL17B			*	
IL18			**	
IL33	****		****	
IL34			*	
TGFB3	***		****	
TNFSF13B	**		*	
TNFSF14			*	
TSLP		*	*	
CXCL1	***		****	
CXCL3	**		***	
CXCL6		**	****	
CXCL8	**		****	
CXCL12		*	****	
CD40	*	*		
CD74			*	
CD200				ns
CTSS	**			
HLA-DRA		**	**	
HLA-DRB1		*	*	
IFIT2			*	
NOTCH3	*			
PTGS1			*	
COL1A2			*	
COL4A1	***		****	
COL5A3		***	****	
FN1	**		*	
LAMA4	**			
LAMA5			*	
TNC			****	
ITGA1		*	***	
ITGA3			*	
LEP	**		****	
MCAM		**	****	
PLEKHA2	*			
BST1				ns
CD34				ns
PDGFRB		*	****	
PDPN	**			
THY1				ns

Enriched in

laminin α4
laminin α5
Collagen

Adj. P-value:

*	<0,05
**	<0,01
***	<0,001
****	<0,0001

Table S2. Selected genes based on the differential expression in FRCs grown on: laminin α4 vs collagen, laminin α5 vs collagen, laminin α4 vs laminin α5.
The differentially expressed genes (DEGs) in FRCs per comparison (laminin α4 vs collagen, laminin α5 vs collagen and laminin α4 vs laminin α5) were determined using DESeq2 with wald test. DEGs for cytokines, chemokines, immune modulatory molecules, extracellular matrix molecules, extracellular matrix-related molecules and FRC surface markers were selected if significant. The comparison in which a DEG was significant are visualized with the colour of the enriched sample: laminin α4 (green), laminin α5 (blue) or collagen (red). These significant DEGs were selected to use in Figure 3, together with four additional genes (grey), for additional characterization of FRCs. Bulk RNAseq data are derived from FRCs between passage 1-4 from donors 1,2 and 3 (collagen: n = 6; laminin α4: n = 6; laminin α5: n = 4). Ns: not significant; *: padj<0,05; ** padj<0,01; ***: padj<0,001; ****: padj<0,0001.

Gene		Laminin α4 vs Collagen	Laminin α5 vs Collagen	Laminin α4 vs laminin α5
CXCL2	Fergusson (2025) Higher in Activated	X		X
CXCL3		X		X
SQSTM1		X		X
SERPINE1		X		
C4orf3	Fergusson (2025) Higher in Control	X	X	X
ARL3		X		X
TRAM2		X		X
COPA		X		X
PJA2		X		X
GAPDH		X		X
TPM4		X		X
CHSY1		X		X
COL4A2		X		X
ARF6		X		X
BMERB1		X		X
COL4A1		X		X
TNS1		X		X
PPP1R35		X		X
PGK1		X		X
MPRIP		X		X
ACTB		X		X
PLP2		X		X
PLOD1		X		X
SPARC		X		X
VKORC1		X		X
ANXA6		X		X
TUBB		X		X
CALD1		X		X
ALDOC		X		X
MEF2C			X	X
COL18A1			X	X
PGF			X	X
DEPP1			X	X
JAG1			X	X
HLA-DRB1			X	X
TSPAN14			X	X
A2M			X	X
SLC29A1			X	X
MCAM			X	X
ESAM			X	X
PHF21A				X
CARD19				X
SH2D3C				X
MZT2B				X
PTEN				X
ANAPC11				X
ALKBH5				X
FEZ2				X
HILPDA				X
LPP				X
IGFBP7				X
TUBA4A				X
INPP5A				X
TACC1				X
ERO1A				X
MRPL43				X
CRIP2				X
IFITM2				X
NOL3				X
ZNF280D				X
TXNIP				X

Gene		Laminin α4 vs Collagen	Laminin α5 vs Collagen	Laminin α4 vs laminin α5
PDLIM1	Fergusson (2025) Higher in Control			X
COL1A2				X
SH3BGRL				X
NR2F2				X
CHCHD10				X
CAV1				X
PDHB				X
IGFBP2				X
MAGEH1				X
FAM162B				X
VWA1				X
AUTS2		X		X
RASIP1		X		X
CSGALNACT1		X		X
CTSH		X		X
TMOD3			X	X
ORMDL1				X
CLPTM1L				X
VAT1				X
PLAT				X
DDOST				X
NSF				X
PPA2				X
ARPC1B				X
ANXA1				X
CFH				X
CIAPIN1				X
ERP29				X
DCN				X
PSMD8		X	X	
SEC24D		X		
PIH1D1		X		
LDHA		X		
MYL6		X		
RBX1		X		
TUBB6		X		
YWHAH		X		
PGAM1		X		
WDR1		X		
NDUFB8		X		
ACTN1		X		
KCTD20		X		
ACTR1A		X		
ACTG1		X		
BCKDK		X		
PPDPF		X		
RRAS		X		
CLEC2B		X		
F11R		X		
SULF2		X		
PIM3		X		
EGFL7		X		
ST6GALNAC3		X		
FGD5		X		
LXN		X		
SEC11C		X		
AP1AR		X		
EBPL		X		
TMEM176B		X		
SELENOP			X	
GNG11			X	

Enriched in
Laminin α4
Laminin α5
Collagen

Table S3. Overlapping differentially expressed genes from FRC bulk RNA sequencing and publicly available single cell RNA sequencing.
Related to Figure 1. List of significantly differentially expressed genes (DEGs) in non-hematopoietic stromal cells between activated and control human LN slices (single cell RNA sequencing from Fergusson *et al*.[S2]) and the DEGs in FRCs per comparison (laminin α4 vs collagen, laminin α5 vs collagen and laminin α4 vs laminin α5) (bulk RNA sequencing). Only the genes enriched in both datasets are visualized. Bulk RNAseq data are derived from FRCs between passage 1-4 from donors 1,2 and 3 (collagen: n = 6; laminin α4: n = 6; laminin α5: n = 4). Colours: laminin α4 (green), laminin α5 (blue) or collagen (red).

Donor	$CD45^+$ (from alive cells)	$CD19^+$ (from $CD45^+$ cells)	$CD11c^+$ (from $CD45^+$ cells)	$CD14^+$ (from $CD45^+$ cells)	$CD3^+$ (from $CD45^+$ cells)	$CD4^+$ (from $CD3^+$ cells)	$CD8^+$ (from $CD3^+$ cells)
Donor 2	99.3%	14.7%	2.7%	0.8%	75.0%	88.7%	2.5%
Donor 4	93.4%	5.4%	1.5%	0.01%	88.8%	98.1%	1.8%
Donor 7	76.1%	4.3%	3.0%	1.2%	85.1%	99.1%	0.9%

Table S4. $CD4^+$ T cell population purity for FRC + T cell co-culture.
Related to Figure 5. After Magnisort $CD4^+$ T cell isolation. As analysed by spectral flow cytometry.

Donor	Sex	Age	LN used for	Used in
1	Male	78 years	FRC culture on LN ECM, collagen and laminin α4	Figure 1, 2, 3, 4<br>Supplemental Figure S1, S2, S3, S4<br>Supplemental Table S1, S2, S3
2	Male	50 years	FRC culture on laminin α5; co-culture FRCs and T cells	Figure 1, 2, 3, 4, 5<br>Supplemental Figure S1, S2, S3, S5, S6, S7, S8, S9<br>Supplemental Table S1, S2, S3, S4
3	Female	39 years	FRC culture on LN ECM, collagen, laminin α4 and laminin α5	Figure 1, 2, 3<br>Supplemental Figure S1, S2, S3<br>Supplemental Table S1, S2, S3
4	Male	53 years	FRC culture on collagen, laminin α4 and laminin α5; co-culture FRCs and T cells	Figure 4, 5<br>Supplemental Figure S5, S7, S8, S9<br>Supplemental Table S4
5	Female	48 years	FRC culture on collagen, laminin α4 and laminin α5	Figure 4
6	Male	71 years	FRC culture on collagen, laminin α4 and laminin α5	Figure 4
7	Female	53 years	co-culture FRCs and T cells	Figure 4, 5<br>Supplemental Figure S5, S7, S8, S9<br>Supplemental Table S4
8	Male	28 years	Immunofluorescence staining	Figure 4
9	Female	48 years	LN ECM isolation	
10	Female	70 years	LN ECM isolation	
11	Male	66 years	LN ECM isolation	
12	Male	65 years	LN ECM isolation	
13	Female	65 years	LN ECM isolation	
14	Female	27 years	LN ECM isolation	
15	Female	73 years	LN ECM isolation	
16	Female	20 years	LN ECM isolation	
17	Female	67 years	LN ECM isolation (2 LNs used)	

Table S5. Characteristics of human lymph node donors.
Related to STAR methods.

Bulk RNAseq sample	Donor	ECM coating	Sorted population
1	1	laminin α4	MHCII^{neg}CD34^{neg}
2	1	laminin α4	MHCII^{+}–CD34^{neg}
3	1	laminin α4	MHCII^{neg}CD34^{+}
4	2	laminin α5	MHCII^{neg}CD34^{neg}
5	2	laminin α5	MHCII^{neg}CD34^{+}
6	1	collagen	MHCII^{neg}CD34^{neg}
7	1	collagen	MHCII^{+}–CD34^{neg}
8	1	Collagen	MHCII^{neg}CD34^{+}
9	1	Native LN ECM	MHCII^{neg}CD34^{neg}
10	1	Native LN ECM	MHCII^{+}–CD34^{neg}
11	1	Native LN ECM	MHCII^{neg}CD34^{+}
15	3	laminin α4	MHCII^{neg}CD34^{neg}
16	3	laminin α4	MHCII^{+}–CD34^{neg}
17	3	laminin α4	MHCII^{neg}CD34^{+}
18	3	laminin α5	MHCII^{neg}CD34^{neg}
19	3	laminin α5	MHCII^{neg}CD34^{+}
20	3	Collagen	MHCII^{neg}CD34^{neg}
21	3	Collagen	MHCII^{+}–CD34^{neg}
22	3	Collagen	MHCII^{neg}CD34^{+}
23	3	Native LN ECM	MHCII^{neg}CD34^{neg}
24	3	Native LN ECM	MHCII^{+}–CD34^{neg}
25	3	Native LN ECM	MHCII^{neg}CD34^{+}

Table S6. Characteristics of bulk RNA sequencing samples.
Related to STAR methods.

References

S1. Grasso, C., Roet, J.E.G., de Graça, C.G., Semmelink, J.F., de Kok, M., Remmerswaal, E., Jongejan, A., Moerland, P.D., Mebius, R.E., and van Baarsen, L.G.M. (2025). Identification and Mapping of Human Lymph Node Stromal Cell Subsets by Combining Single-Cell RNA Sequencing with Spatial Transcriptomics. Eur J Immunol 55, e51218. 10.1002/eji.202451218.

S2. Fergusson, J.R., Siu, J.H.Y., Gupta, N., Jenkins, E., Nee, E., Reinke, S., Ströbel, T., Bhalla, A., Kandage, S.M., Courant, T., et al. (2025). Ex vivo model of functioning human lymph node reveals role for innate lymphocytes and stroma in response to vaccine adjuvant. Cell Rep 44, 115938. 10.1016/j.celrep.2025.115938.
